# MurA-catalyzed synthesis of 5-enolpyruvylshikimate-3-phosphate confers glyphosate tolerance in bryophytes

**DOI:** 10.1073/pnas.2412997121

**Published:** 2024-11-11

**Authors:** Samuel Caygill, Thomas Köcher, Liam Dolan

**Affiliations:** ^a^Gregor Mendel Institute, Vienna BioCenter, Vienna 1030, Austria; ^b^Department of Biology, University of Oxford, Oxford OX1, United Kingdom; ^c^Vienna BioCenter Core Facilities, Vienna BioCenter, Vienna 1030, Austria

**Keywords:** glyphosate, shikimate pathway, enolpyruvyl transferase, EPSPS, MurA

## Abstract

Glyphosate is a broad-spectrum herbicide that kills most vascular plant weeds but is ineffective against many bryophytes. Glyphosate competitively inhibits the enolpyruvyl transferase enzyme 5-enolpyruvylshikimate-3-phosphate synthase (EPSPS). EPSPS catalyzes the production of 5-enolpyruvylshikimate-3-phosphate (EPSP)—an intermediate in the shikimate pathway—from shikimate-3-phosphate (S3P) and phosphoenolpyruvate (PEP) substrates. Here, we show that *Marchantia polymorpha* mutants with loss-of-function mutations in a closely related enolpyruvyl transferase, Mp*MurA,* were more sensitive to glyphosate than wild-type controls. Overexpression of Mp*MurA* in *M. polymorpha* increased glyphosate tolerance, and heterologous expression of the *M. polymorpha* MurA enzyme in *Arabidopsis thaliana* conferred glyphosate resistance. Furthermore, we demonstrate that MpMurA catalyzes the production of EPSP from S3P and PEP substrates. These data demonstrate that MpMurA contributes to glyphosate tolerance in *M. polymorpha*. We speculate that the existence of two independent mechanisms for EPSP synthesis—one canonical and EPSPS-dependent, and the other MurA-dependent—may account for glyphosate tolerance in bryophytes. Alternatively, MurA in bryophytes may bind glyphosate, thereby leaving more unbound EPSPS enzymes available, allowing aromatic amino acid biosynthesis to continue.

## Significance

Glyphosate accounts for approximately 20% of herbicides sprayed globally. Glyphosate targets the enolpyruvyl transferase enzyme 5-enolpyruvylshikimate-3-phosphate synthase (EPSPS), which is involved in the biosynthesis of aromatic amino acids. One of the two monophyletic groups of land plants, the bryophytes, are tolerant to glyphosate. We show in *Marchantia polymorpha* that this is the result of a second enolpyruvyl transferase enzyme, MurA. Many angiosperms do not encode this enzyme. We find that MurA can catalyze the same reaction as the glyphosate target EPSPS. Our finding of a catalytic step in aromatic amino acid synthesis has the potential to be the basis of a source of glyphosate tolerance in weeds and crops and contribute to strategies for herbicide discovery.

Glyphosate has been extensively used to control the growth of vascular plant weeds in agricultural, urban, and domestic environments and is the most used herbicide globally (1). Glyphosate treatment leads to plant death after 4 to 20 d (2, 3). Glyphosate competitively inhibits 5-enolpyruvylshikimate-3-phosphate synthase (EPSPS) that catalyzes the reaction between substrates shikimate-3-phosphate (S3P) and phosphoenolpyruvate (PEP), to produce 5-enolpyruvylshikimate-3-phosphate (EPSP) and phosphate, a key step in the shikimate pathway, which precedes aromatic amino acid biosynthesis. EPSP is therefore an essential intermediate in aromatic amino acid biosynthesis. While glyphosate is known to inhibit EPSPS, the exact cause of glyphosate-induced death in plants is unknown. S3P accumulates when sensitive plants are treated with glyphosate, which is then dephosphorylated to shikimate (4–6). Lower levels of shikimate accumulate in glyphosate-treated plants that have evolved resistance or are naturally tolerant to glyphosate (7–11). Consequently, shikimate accumulation levels following glyphosate treatment have become a useful biomarker to indicate glyphosate sensitivity or resistance (11–13). An essential role of EPSPS in aromatic amino acid biosynthesis accounts for the glyphosate sensitivity of vascular plants. However, there is anecdotal evidence from the horticultural literature that glyphosate does not effectively control bryophyte weeds (14–16). The mechanism of glyphosate tolerance in bryophytes is unknown.

Genes encoding EPSPS—the target of glyphosate—are present in all lineages of streptophytes. EPSPS belongs to the small family of enolpyruvyl transferase enzymes comprising two enzymes, EPSPS and uridine diphosphate (UDP) *N*-acetylglucosamine enolpyruvyl transferase (MurA). Both enzymes are present in most bryophytes, while MurA is absent from many angiosperms (17–20). MurA catalyzes the first step in peptidoglycan biosynthesis and, like EPSPS, MurA catalyzes the transfer of an enolpyruvyl moiety from phosphoenolpyruvate (PEP) but in this case the receiver is the 3’-hydroxyl group of UDP *N*-acetylglucosamine (UDP-GlcNAc) (21). Furthermore, MurA and EPSPS are structurally similar, described as an “inside-out α/β-barrel” (22–24). Therefore, we hypothesized that MurA in bryophytes also catalyzes the reaction between PEP and S3P to form EPSP, and that the presence of an alternative enzyme for this reaction would confer glyphosate tolerance in bryophytes.

To test the hypothesis that MurA catalyzes the production of EPSP and confers glyphosate tolerance, we characterized *Marchantia polymorpha* mutants with altered levels of MpMurA activity. We showed that loss-of-function mutations in the *MurA* gene increased glyphosate sensitivity, while overexpression of MpMurA increased glyphosate tolerance. Furthermore, heterologous expression of the *M. polymorpha* enzyme in *Arabidopsis thaliana* conferred glyphosate resistance. We demonstrated that MpMurA catalyzes the transfer of the enolpyruvyl moiety of PEP to S3P to produce EPSP. These findings are consistent with glyphosate tolerance in *M. polymorpha* resulting from two independent mechanisms—one EPSPS-dependent and the other MurA-dependent—to produce EPSP, an essential aromatic amino acid biosynthesis intermediate. This finding is also consistent with an alternative hypothesis, whereby the active sites of MurA and EPSPS are sufficiently similar to allow MurA to bind glyphosate, and in doing so, prevent glyphosate from inhibiting EPSPS.

## Results

### Bryophytes are Glyphosate Tolerant.

**Fig. 1.**
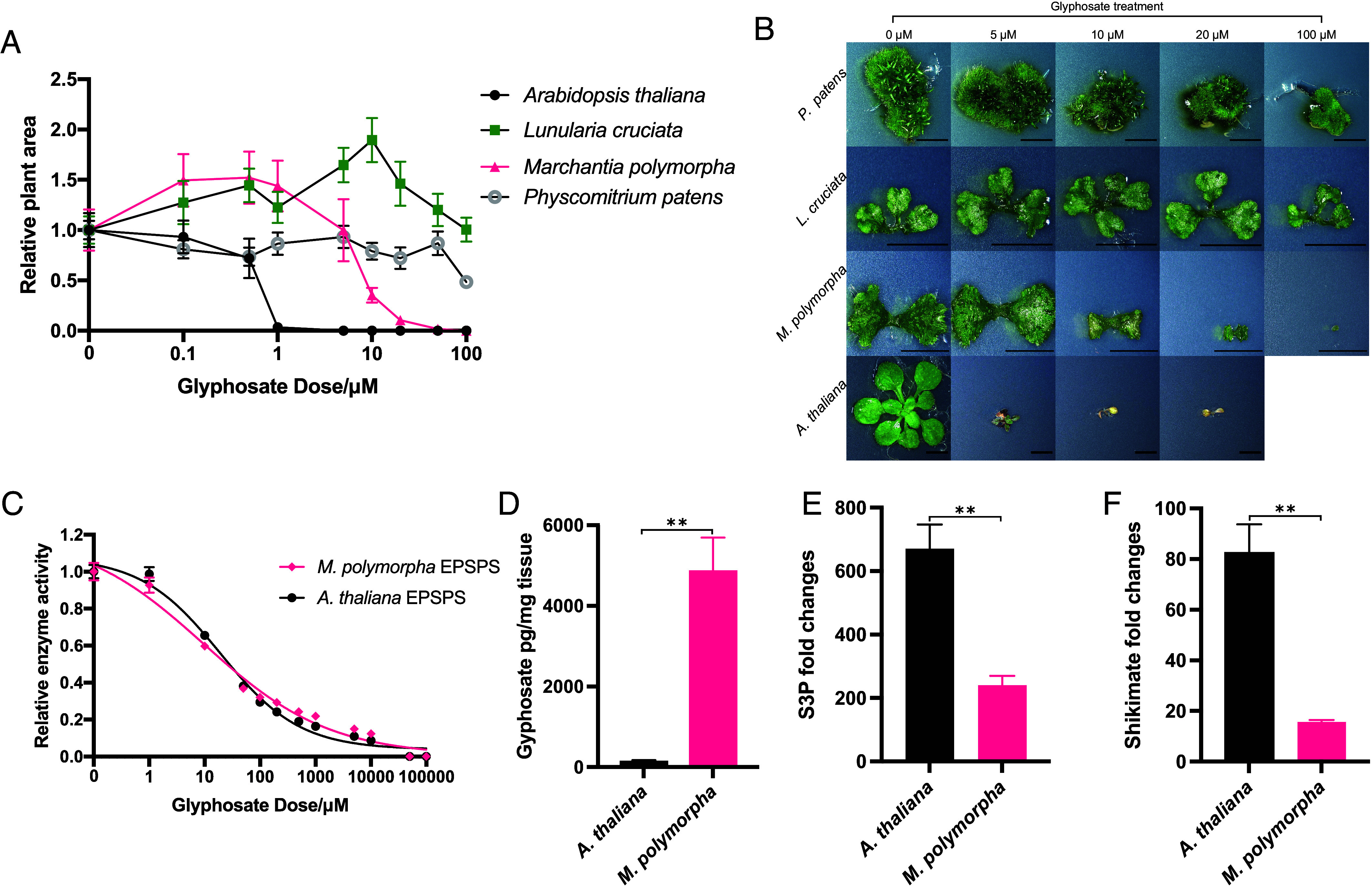
Bryophytes are tolerant to glyphosate while angiosperms are not (*A*) Dose–responses of the plant areas of *A. thaliana*, *M. polymorpha, L. cruciata* and *P. patens* plants grown for 21-d on glyphosate treated plates. Error bars are ±SE. (*B*) Brightfield images of individual plants from each species on 5 selected doses of glyphosate taken with a Keyence VHX-7000 microscope. No *A. thaliana* plant germinated at 100 μM glyphosate. (Scale bar, 5 mm.) (*C*) Dose–response curves of relative activities of purified EPSPS enzymes from *A. thaliana* and *M. polymorpha* at varying glyphosate concentrations. The data are fitted with a four-parameter log-logistic regression curve. Error bars are ±SE. (*D*) Absolute values of glyphosate in the tissues of 14-d-old *A. thaliana* and *M. polymorpha* plants, measured using LC-MS/MS, after treatment with 5 μM of glyphosate for 2 d. Error bars are ±SE. (*E*) Shikimate and (*F*) S3P ion counts were measured in *A. thaliana* and *M. polymorpha* plants treated with 5 μM glyphosate for 2 d using LC-MS/MS, and the fold changes calculated. Error bars represent SE. Statistical significances are based on Welch’s *t* tests. **P < 0.001, ns = not significant.

Extensive anecdotal evidence demonstrates that bryophytes are tolerant to glyphosate. To experimentally confirm this observation, we compared the glyphosate sensitivity of three bryophyte species—*M. polymorpha, Lunularia cruciata* and *Physcomitrium patens*—with the glyphosate sensitive angiosperm, *A. thaliana* (Fig. 1 *A* and *B*). Each of the bryophytes was more tolerant to glyphosate than *A. thaliana*. Furthermore, growth was minimally affected in both *L. cruciata* and *P. patens* across all glyphosate concentrations tested (Fig. 1 *A* and *B*). These data are consistent with the observation that bryophytes are more glyphosate tolerant than angiosperms.

We set out to define the mechanism of glyphosate tolerance in bryophytes, using *M. polymorpha* as an experimental system. First, we tested the hypothesis that *M. polymorpha* encodes a tolerant version of the EPSPS enzyme targeted by glyphosate. We conducted a glyphosate dose–response experiment comparing the activities of purified recombinant EPSPS proteins from *M. polymorpha* and *A. thaliana* in vitro. The dose–response relationship of the EPSPS enzymes from *M. polymorpha* and *A. thaliana* to glyphosate was similar (Fig. 1*C*). The IC_50_ values for glyphosate were 9.60 μM (SE = ± 9.12) for *M. polymorpha* EPSPS (MpEPSPS) and 14.49 μM (SE = ±5.98) for *A. thaliana* EPSPS (AtEPSPS). These values are not significantly different (Welch’s *t* test, *P* > 0.05). The Michaelis–Menten affinity constants (K_m_) of AtEPSPS and MpEPSPS for the substrate S3P differ slightly with values of 0.1967 mM (SE = ± 0.04463) and 0.1197 mM (SE = ± 0.01282), respectively (*SI Appendix*, Fig. S1 *A* and *B*). The K_m_ values for the substrate PEP, with which glyphosate competes for binding to EPSPS, of MpEPSPS and AtEPSPS were similar at 0.180 mM (SE = ±0.026) and 0.163 mM (SE = ±0.034), respectively (*SI Appendix*, Fig. S1 *C* and *D*). Maximum enzyme velocity (V_max_) of AtEPSPS was 3.43-fold higher for PEP and 4.26-fold higher for S3P than MpEPSPS, corresponding to higher catalytic constant (K_cat_) and catalytic efficiency (K_cat_/K_m_) of AtEPSPS compared to MpEPSPS (*SI Appendix*, Fig. S1*E*). These differences suggest that AtEPSPS is more tolerant to glyphosate than MpEPSPS. Therefore, differences in *M. polymorpha* EPSPS enzyme kinetics and sensitivity to glyphosate cannot account for glyphosate tolerance in *M. polymorpha*.

Another hypothesis is that *M. polymorpha* is tolerant to glyphosate because the herbicide is not absorbed by the plants. To test whether *M. polymorpha* takes up glyphosate, the quantity of glyphosate in *M. polymorpha* thalli and *A. thaliana* leaves (positive control), from plants grown on media supplemented with 5 μM glyphosate, was measured using LC-MS/MS (Fig. 1*D*). Glyphosate levels were higher in *M. polymorpha* than in *A. thaliana* after 2 d of 5 µM glyphosate treatment. Despite *A. thaliana* being sensitive to 5 μM glyphosate (Fig. 1 *A* and *B*), it accumulated roughly 36 times less glyphosate than *M. polymorpha.* These data demonstrate that glyphosate is taken up by *M. polymorpha* and indicate that glyphosate tolerance in *M. polymorpha* cannot be explained by low glyphosate uptake. Therefore, an alternative mechanism must confer glyphosate tolerance in *M. polymorpha*.

The inhibition by glyphosate of the EPSPS-catalyzed reaction between S3P and PEP blocks the shikimate pathway. It has been reported that S3P accumulates and is rapidly dephosphorylated to shikimate in sensitive, glyphosate-treated plants (4, 5, 25). To test whether shikimate and S3P accumulate following glyphosate treatment in *M. polymorpha,* we measured the quantities of shikimate (Fig. 1*E*) and S3P (Fig. 1*F*) in the tissues of *A. thaliana* (positive control) and *M. polymorpha* grown on media supplemented with 5 μM glyphosate using LC-MS/MS. In untreated control samples, shikimate levels were higher in *M. polymorpha* than *A. thaliana* (*SI Appendix*, Fig. S1*G*). Shikimate ion counts were also measured after plants were grown on media supplemented with 5 μM glyphosate. After 2 d of glyphosate treatment, the fold increase of shikimate was approximately 5 times greater in *A. thaliana* (77.83-fold) than in *M. polymorpha* (15.75-fold) compared to untreated plants. A lower accumulation of shikimate therefore correlates with glyphosate tolerance in *M. polymorpha* (Fig. 1*E*). S3P also accumulated more in *A. thaliana* (670.90-fold) than in *M. polymorpha* (240.16-fold) following glyphosate treatment (Fig. 1*F*). There were no differences in the quantities of S3P in the tissues of *A. thaliana* and *M. polymorpha* in untreated control samples (*SI Appendix*, Fig. S1 *H* and *I*). These data indicate that the greater sensitivity of *A. thaliana* to glyphosate is correlated with a greater relative increase in S3P and shikimate levels following glyphosate treatment than in *M. polymorpha*. The increase in shikimate in *A. thaliana* is likely the result of accumulated S3P being dephosphorylated into shikimate. The accumulation of lower S3P levels on glyphosate treatment in *M. polymorpha* than in *A. thaliana*, suggests that this intermediate is being metabolized in the liverwort, but not in the angiosperm. Therefore, we hypothesized the presence of an additional enzyme that metabolizes S3P in *M. polymorpha* but not in *A. thaliana*.

### MurA Is Derived from an EPSPS-Like Ancestral Protein.

EPSPS and MurA enzymes each transfer the enolpyruvyl moiety from PEP to the substrates S3P and UDP-GlcNAc, respectively (24, 26–28). Both proteins are encoded in the *M. polymorpha* genome. By contrast, EPSPS, but not MurA, is encoded in the *A. thaliana* genome. Therefore, we hypothesized that the presence of the structurally and catalytically similar MurA enzyme in *M. polymorpha* confers glyphosate tolerance in *M. polymorpha*.

**Fig. 2.**
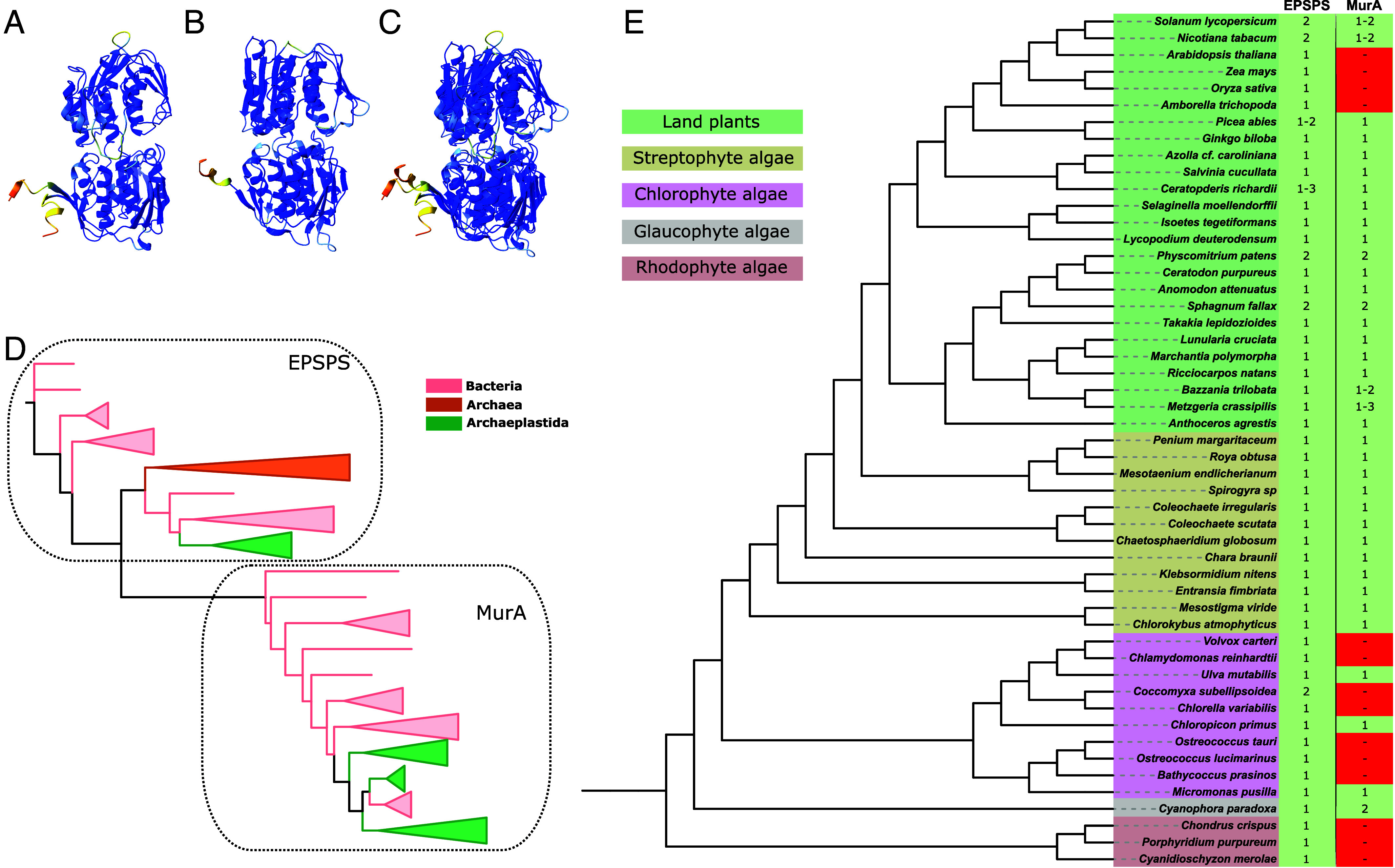
EPSPS and MurA are closely related and structurally similar enolpyruvyl-transferase enzymes which are highly conserved among the Archaeplastida. (*A*) AlphaFold prediction of the EPSPS enzyme from *M. polymorpha*. (*B*) AlphaFold prediction of the MurA enzyme from *M. polymorpha*. (*C*) Overlay of the EPSPS and MurA enzymes from *M. polymorpha* produced in UCSF ChimeraX using the matchmaker function. AlphaFold per residue confidence score (pLDDT) ranging from 0 and 100. Color denotes confidence in the prediction: Dark blue = Very high (pLDDT > 90); Light blue = Confident (90 > pLDDT > 70); Yellow = Low (70 > pLDDT > 50); Orange = Very low (pLDDT < 50). (*D*) Phylogenetic tree based on Maximum Likelihood statistics with collapsed clades representing the evolution of EPSPS and MurA based on the full tree presented in *SI Appendix*, Fig. S2. (*E*) Species tree based on (29–31). Presence or absence of EPSPS and MurA in each species was determined using BLASTp searches with the amino acid sequence of each enzyme from *M. polymorpha* as a query. Green depicts presence, red absence, and the number of enzyme paralogs when present is given.

AlphaFold predicts that MpEPSPS and MpMurA proteins are structurally similar, consistent with the hypothesis that MurA and EPSPS are functionally similar (Fig. 2 *A* and *B*). Each enzyme consists of two domains comprising α-helices and β-sheets joined by a flexible linker region. The cleft between the 2 domains forms the active site of each protein (27). The RMSD of atomic positions of the aligned protein structures was 1.347 Å for 146 pruned atom pairs (14.648 Å for all 451 pairs) (Fig. 2*C*). This indicates a high degree of structural similarity between MpMurA and MpEPSPS. The percent identity of the amino acid sequences of MpMurA and MpEPSPS was 20.34% identity, indicating a moderate level of sequence similarity between MpMurA and MpEPSPS. In comparison, the RMSD of the aligned AlphaFold protein structures of *M. polymorpha* EPSPS and *A. thaliana* EPSPS was 0.435 Å for 427 pruned atom pairs (0.915 Å for all 439 pairs). The percent identity of *A. thaliana* and *M. polymorpha* EPSPS proteins was higher at 61.61%.

To investigate the evolutionary history of enolpyruvyl transferase enzymes we first identified EPSPS sequences in bacteria, archaea, and Archaeplastida genomes, and MurA sequences were found in bacteria and Archaeplastida, but absent from archaea (Dataset S1). Using these sequences we generated a multiple sequence alignment of 79 enolpyruvyl transferase enzyme sequences (Dataset S2). The alignment was used to construct a Maximum Likelihood tree (*SI Appendix*, Fig. S2), summarized in Fig. 2*D*. The topology of the tree shows that MurA sequences and many EPSPS sequences share a common ancestor and that extant MurA enzymes evolved from an EPSPS-like ancestor among bacteria. A monophyletic EPSPS clade including bacteria, archaea, and Archaeplastida sequences and a monophyletic MurA clade including bacteria and Archaeplastida sequences, together form a well-supported, monophyletic clade that is sister to a polyphyletic clade of bacterial EPSPS enzymes. These data support the hypothesis that MurA enzymes evolved in bacteria from an EPSPS-like ancestral enzyme.

At least one EPSPS was encoded in each of the sampled Archaeplastida genomes. By contrast, 37 of the 51 sampled Archaeplastida genomes encoded a MurA enzyme. While a *MurA* gene was present in the genomes of all sampled nonangiosperm streptophytes and the only glaucophyte sampled, only 2 of the 6 sampled angiosperms, 3 of the 10 chlorophyte algae, and none of the 3 rhodophyte algae encoded a MurA enzyme (Fig. 2*E*). To estimate the proportion of angiosperms encoding a *MurA* gene, a BLASTp search of the 857 angiosperm species included in the 1,000 plants (1KP) transcriptomes project (29), showed that approximately 20% (178/857) of these angiosperms encode a version of MurA. By contrast, almost 100% of the 441 (440/441) nonangiosperm streptophytes in that database encode a MurA enzyme. An extensive analysis of angiosperm MurA enzymes can be found in *SI Appendix*, Fig. S3. These data suggest that *MurA* was present in the last common ancestor of the Archaeplastida and has been lost multiple times since. Furthermore, there have been numerous losses of the *MurA* gene within the angiosperm lineage, and expression of *MurA* in glyphosate-sensitive angiosperms which have retained *MurA*, such as *Solanum tuberosum* and *Nicotiana attenuata*, is very low compared to *EPSPS*, especially in the leaves (*SI Appendix*, Fig. S3 *G* and *H*). For example, in *N. attenuate, MurA* is expressed 100-fold less than *EPSPS* in the leaves, while *MurA* is not expressed at all in the leaves of *S. tuberosum*. In contrast, *MurA* is expressed 10-fold less than *EPSPS* in *M. polymorpha*, and there is consistent expression in most tissues (32), (*SI Appendix*, Fig. S3*I*). Therefore, the high structural similarity of EPSPS and MurA in *M. polymorpha* and absence of MurA from most glyphosate-sensitive plants is consistent with the involvement of MurA in glyphosate tolerance in *M. polymorpha*.

### Mp*MurA* Confers Glyphosate Tolerance in *M. polymorpha* and Glyphosate Resistance in *A. thaliana*.

**Fig. 3.**
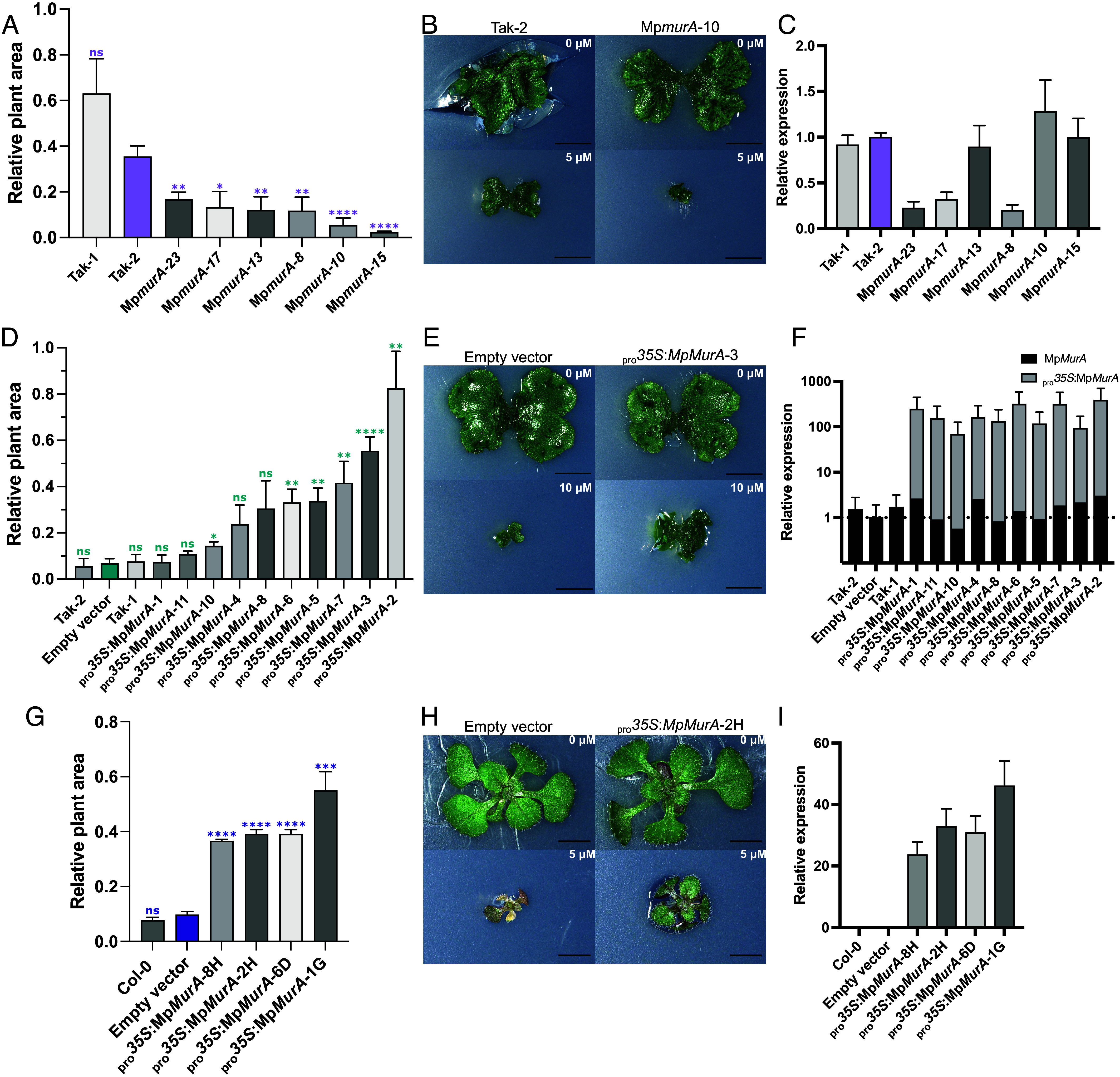
Mp*murA* loss-of-function mutants are more sensitive to glyphosate and overexpression of MpMurA causes an increase in resistance to glyphosate. (*A*) Plant area ratios of *M. polymorpha* wild-type (Tak-1 and Tak-2) and Mp*murA* loss-of-function lines grown on 0 μM and 5 μM of glyphosate for 14 d. The autofluorescing areas were measured and the ratios between the treated and untreated plants calculated and plotted. Error bars are ±SE. (*B*) Brightfield images of individual Tak-2 and Mp*murA*-10 plants on control and 5 μM glyphosate media taken with a Keyence VHX-7000 microscope. (Scale bar, 5 mm.) (*C*) Relative expression of Mp*MurA* in *M. polymorpha* Mp*murA* lines compared to Tak-2 calculated from fold differences in mRNA abundance. (*D*) Plant area ratios of *M. polymorpha* wild-type and Mp*MurA* overexpression lines. Plants were grown on 0 μM and 10 μM of glyphosate and the autofluorescing areas used to measure the ratio between treated and untreated plants. Error bars are ±SE. (*E*) Brightfield images of individual Empty vector control and _pro_*35S*:Mp*MurA*-3 plants on control and 10 μM glyphosate media taken with a Keyence VHX-7000 microscope. (Scale bar, 5 mm.) (*F*) Relative expression of Mp*MurA* and _pro_*35S*:Mp*MurA* in *M. polymorpha* Mp*MurA* overexpression lines compared to Mp*MurA* expression in the empty vector control, calculated from fold differences in mRNA abundances. (*G*) Plant area ratios of *A. thaliana* wild-type and *A. thaliana* lines expressing Mp*MurA* grown on 0 μM and 5 μM of glyphosate for 14 d. The autofluorescing areas were measured and the ratios between the treated and untreated plants calculated and plotted. Error bars are ±SE. (*H*) Brightfield images of individual Empty vector control and _pro_*35S*:Mp*MurA*-2H plants on control and 5 μM glyphosate media taken with a Keyence VHX-7000 microscope. (Scale bar, 2.5 mm.) (*I*) Relative expression of Mp*MurA* in _pro_*35S*:Mp*MurA A. thaliana* lines compared to the expression of *UBC21*, calculated from fold differences in mRNA abundance. All tests of statistical differences were Brown–Forsythe and Welch ANOVA performed using Tak-2 or empty vector control plant area ratios as the baseline: *P < 0.05, **P < 0.01, ***P < 0.001, ****P < 0.00001, ns = not significant.

To functionally test whether MpMurA contributes to glyphosate tolerance in *M. polymorpha*, we genetically manipulated the expression of Mp*MurA*. We generated Mp*murA* loss-of-function mutants by CRISPR-Cas9 mutagenesis of sporeling generated from a cross between Takaragaike-1 (Tak-1) and Takaragaike-2 (Tak-2) plants. Six putative Mp*murA* mutants were isolated, with a variety of genotypes ranging from substitutions of two base pairs to large deletions which are predicted to result in the expression of a truncated version of the MpMurA protein (*SI Appendix*, Fig. S4 *A* and *B*). The growth rate of three putative Mp*murA* mutants, Mp*murA*-8, Mp*murA*-10, and Mp*murA*-15, was not significantly different from the wild-type accessions Tak-1 and Tak-2, despite a predicted loss of function of MurA (*SI Appendix*, Fig. S4*C*). To test the hypothesis that MurA contributes to glyphosate tolerance, we compared the glyphosate sensitivity of Mp*murA* loss-of-function mutants to wild-type plants (Fig. 3 *A* and *B*). Tak-2 wild-type plants are more sensitive to glyphosate than Tak-1 and were therefore used as a conservative baseline for statistical comparison between Mp*murA* mutants and wild-type plants. All Mp*murA* mutants were more sensitive to glyphosate than Tak-2 (Fig. 3*A*; Brown–Forsythe and Welch ANOVA, *P* < 0.05). However, none of the mutants were more sensitive to the branched-chain amino acid inhibitor chlorsulfuron (*SI Appendix*, Fig. S4*C*), showing the increase in sensitivity was specific to glyphosate. We also measured the relative expression of Mp*MurA* in Mp*murA* mutants using RT-qPCR. Steady state levels of the Mp*MurA* transcript were lower in Mp*murA*-23, Mp*murA*-17, and Mp*murA*-8 lines than in wild type, while steady state levels of the Mp*MurA* transcript were similar or slightly higher in Mp*murA*-13, Mp*murA*-10, and Mp*murA*-15 lines than wild type (Fig. 3*C*). We conclude that mutants with loss-of-function mutations in the Mp*MurA* gene are more sensitive to glyphosate than wild-type plants.

To test whether increased Mp*MurA* expression confers increased glyphosate tolerance, we generated Mp*MurA* overexpression lines in *M. polymorpha* and compared their sensitivity to control plants transformed with an empty vector, and untransformed wild type (Tak-1 or Tak-2) (Fig. 3 *D* and *E*). The growth of plants overexpressing Mp*MurA,* under the transcriptional control of the *CaMV35S* promoter (_pro_*35S:*Mp*MurA*), on 10 μM glyphosate was greater than untransformed Tak-1 and Tak-2 plants or plants overexpressing YFP (empty vector controls, _pro_*35S:YFP*) (Fig. 3 *D* and *E*). The steady state levels of MpMurA mRNA were higher in all lines transformed with the _pro_*35S:*Mp*MurA* construct than wild-type controls (Fig. 3*F*). Therefore, loss of Mp*MurA* function makes *M. polymorpha* more sensitive to glyphosate than wild type, while Mp*MurA* overexpression increases tolerance. These results are consistent with the hypothesis that Mp*MurA* contributes to glyphosate tolerance in *M. polymorpha*.

To test independently if MpMurA confers glyphosate tolerance, we expressed the bryophyte MpMurA enzyme in the angiosperm *A. thaliana* (_pro_*35S:*Mp*MurA*). Plants transformed with the _pro_*35S:*Mp*MurA* construct were consistently more resistant to glyphosate than the empty vector controls (Fig. 3 *G* and *H*). Furthermore, the steady state levels of Mp*MurA* mRNA in *A. thaliana* plants transformed with the _pro_*35S:*Mp*MurA* construct correlated with the magnitude of glyphosate resistance conferred. In conclusion, heterologous expression of Mp*MurA* confers glyphosate resistance in *A. thaliana*.

### The Role of MurA in Glyphosate Tolerance in *M. polymorpha* Is Independent of Its Function in Chloroplasts.

**Fig. 4.**
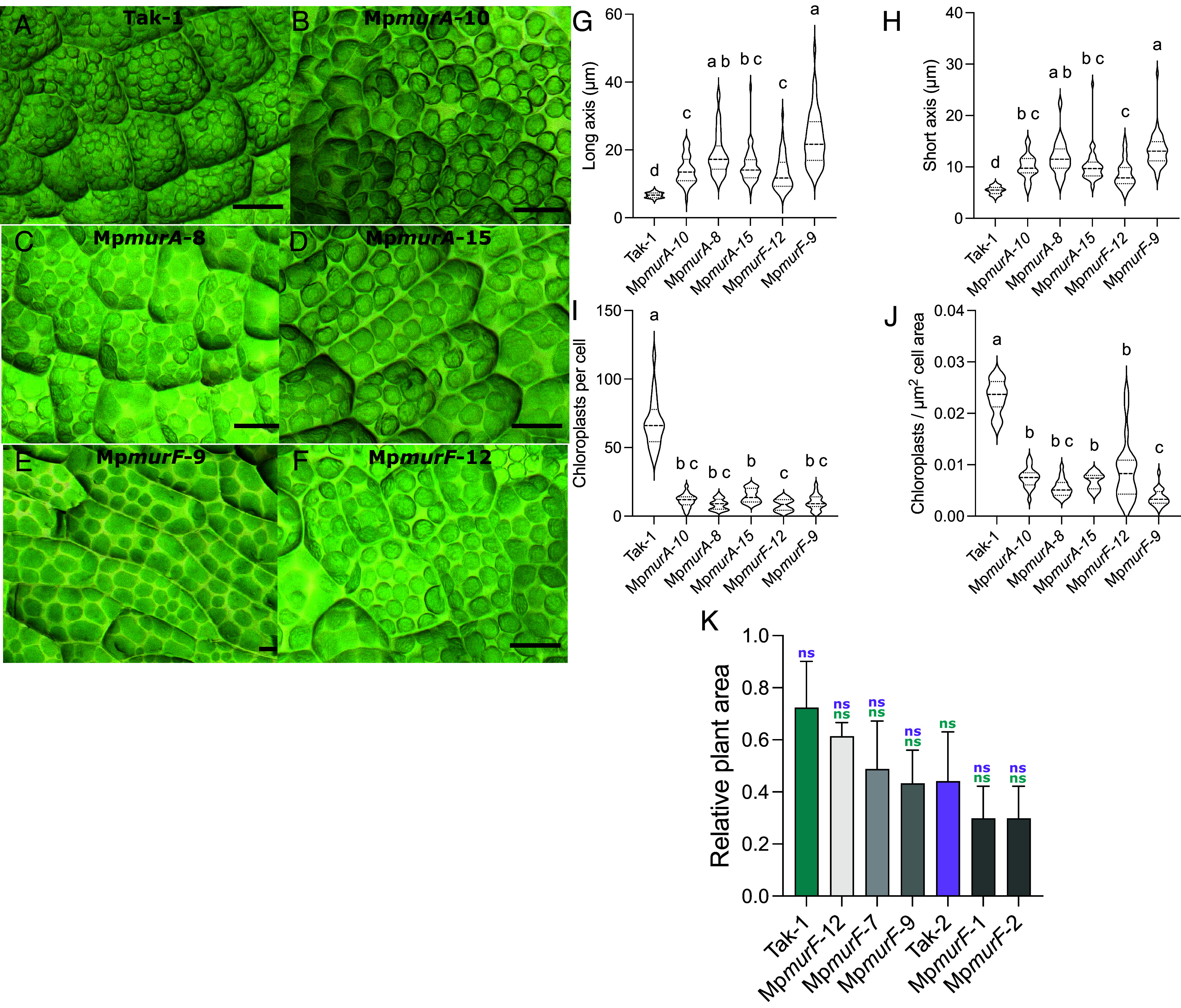
Loss-of-function of MurF and MurA proteins in *M. polymorpha* both cause a macrochloroplast phenotype, but there is no change in glyphosate sensitivity in *murF* mutants. (*A*–*F*) Stereomicroscope images of chloroplasts in 3-d-old gemmae from *M. polymorpha* wild-type (Tak-1) (*A*), Mp*murA*-10 (*B*), Mp*murA*-8 (*C*), Mp*murA*-15 (*D*), Mp*murF*-9 (*E*), and Mp*murF*-12 (*F*) lines. (Scale bar, 25 μm.) (*G*–*J*) Violin plots of chloroplast and cell measurements of wild-type (Tak-1) and Mp*murA* and Mp*murF* loss-of-function mutants calculated using the image analysis software FIJI. The chloroplast longest axis (*G*), shortest axis (*H*), chloroplast abundance per cell (*I*), and chloroplast abundance per unit area of cell (*J*) were measured or calculated. A nonparametric Kruskal–Wallis multiple comparison test was performed to compare values between mutants and Tak-1. Different letters indicate significant differences (*P* < 0.05). (*K*) *M. polymorpha* wild-type (Tak-1 and Tak-2) and Mp*murF* loss-of-function lines were grown on 0 μM and 5 μM of glyphosate. The autofluorescing areas were measured and the ratio between the treated and untreated plants calculated and plotted. Error bars are ±SE. Brown–Forsythe and Welch ANOVA tests were performed using Tak-1 and Tak-2 relative plant area ratios as baselines. ns = not significant.

The target of glyphosate, EPSPS, is chloroplast localized. Disruption of MurA function causes a macrochloroplast phenotype and defects in chloroplast division (18–20, 33). Therefore, it is formally possible that chloroplast defects in *M. polymorpha murA* loss-of-function mutants confer sensitivity to glyphosate. To test the hypothesis that MurA contributes to glyphosate tolerance in *M. polymorpha*, independently of its role in regulating chloroplast size and division, we generated Mp*murF* loss-of-function mutant *M. polymorpha* plants using CRISPR-Cas9. MurF is an essential enzyme in peptidoglycan biosynthesis, like MurA. It catalyzes the formation of UDP-MurNAc-pentapeptide by adding D-alanyl-D-alanine to an acceptor molecule (34, 35). We generated five putative MpmurF loss-of-function mutants using CRISPR (*SI Appendix*, Fig. S5 *A* and *B*). Both Mp*murF* and Mp*murA* loss-of-function mutants developed larger and fewer chloroplasts than wild-type plants, consistent with the related roles of MpMurF and MpMurA in chloroplast peptidoglycan formation (Fig. 4 *A*–*J*). Chloroplast size and abundance differed little among Mp*murA* loss-of-function mutants. However, the long axis of chloroplasts from Mp*murA*-10 is shorter than those from Mp*murA*-8 and Mp*murA*-15 (*P* < 0.05). The chloroplast size and abundance parameters of Mp*murF* loss-of-function mutants differed from each other in all cases except chloroplasts per μm^2^ cell area (*P* < 0.05).

If Mp*murF* mutants and wild type were equally sensitive to glyphosate, and more tolerant than Mp*murA* mutants, it would support the hypothesis that MpMurA confers glyphosate tolerance in *M. polymorpha* through a mechanism independent of its function in peptidoglycan biosynthesis. The glyphosate sensitivity of Mp*murF* loss-of-function mutants was not significantly different from Tak-1 and Tak-2 wild-type plants (Brown–Forsythe and Welch ANOVA, *P* > 0.05) (Fig. 4*K*). Furthermore, the dose–response relationships to glyphosate of lines Mp*murF*-2 and Mp*murF*-12 are indistinguishable from wild-type plants and other Mp*murF* mutants (*SI Appendix*, Fig. S5*C*). These data indicate that MpMurA confers glyphosate tolerance through a mechanism independent of the role in chloroplast peptidoglycan biosynthesis it shares with MpMurF.

### MpMurA Catalyzes the Same Reaction as MpEPSPS.

**Fig. 5.**
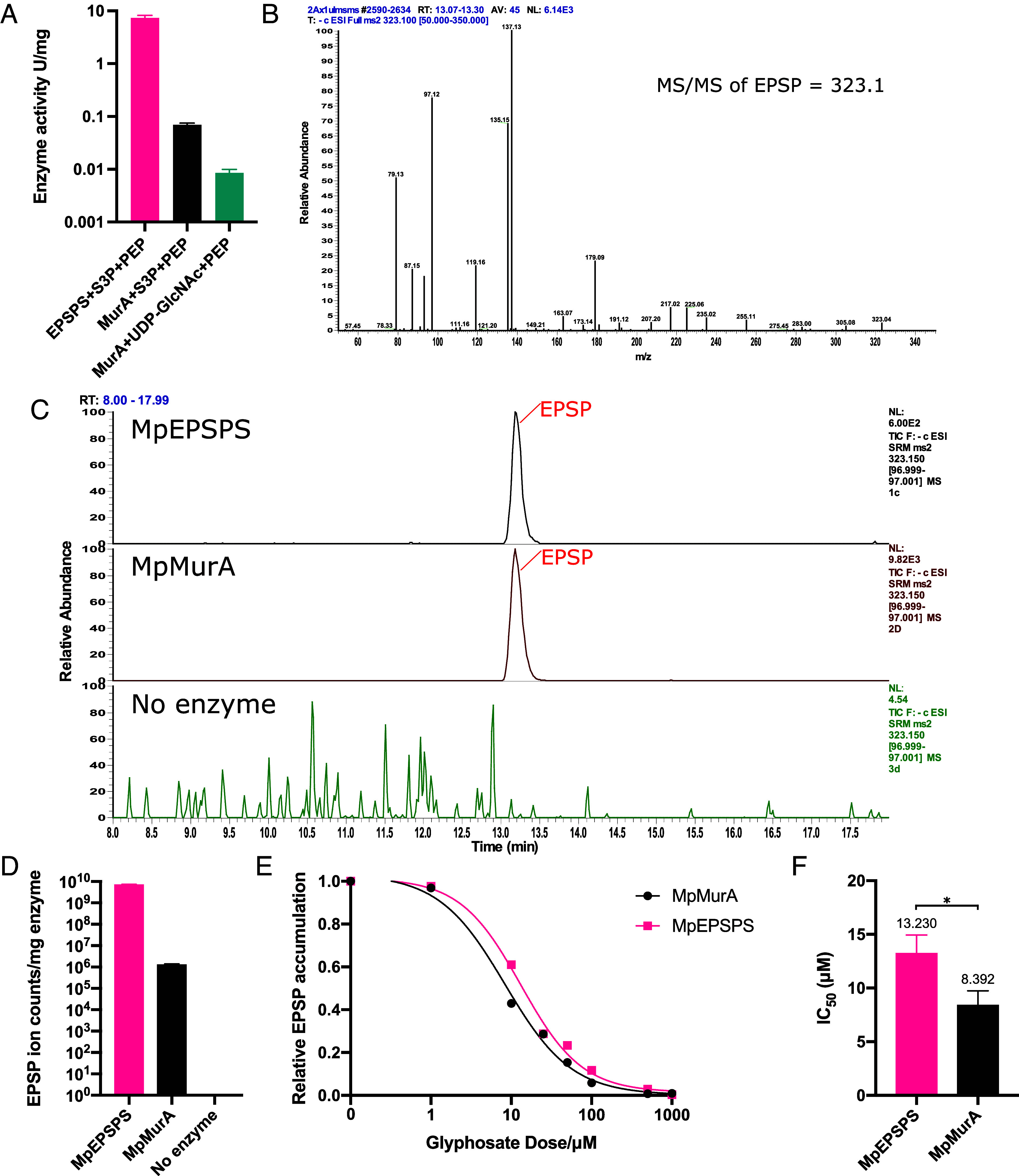
MpMurA catalyzes the reaction between shikimate-3-phosphate and phosphoenolpyruvate to produce 5-enolpyruvylshikimate-3-phosphate. (*A*) Enzymatic activity of MpEPSPS and MpMurA with the substrates S3P and PEP and of MpMurA with UDP-GlcNAc and PEP measured as μmol phosphate produced per minute of reaction time per mg of enzyme (U/mg). (*B*) MS/MS spectrum of the precursor ion *m/z* 323.1 in the negative ion mode. Using a collision energy of 20 eV, we observed fragments at *m/z* 79 and *m/z* 97, characteristic of phosphorylated metabolites. (*C*) SRM chromatograms of EPSP assayed in reaction mixes containing S3P and PEP with MpEPSPS, MpMurA, and no enzyme. EPSP was only identified when either MpEPSPS or MpMurA was present. (*D*) Quantity of EPSP measured with LC-MS/MS when S3P and PEP were incubated with MpEPSPS, MpMurA, and no enzyme. (*E*) Dose–response curve of the relative accumulation of EPSP measured by LC-MS/MS when S3P and PEP were incubated with MpEPSPS and MpMurA along with varying concentrations of glyphosate. The data are fitted with a four-parameter log-logistic regression curve. (*F*) The IC_50_ of MpMurA for glyphosate was significantly lower than that of MpEPSPS (Welch’s *t* test, *P* = 0.0445).

Since putative Mp*murA* loss-of-function mutants are more sensitive to glyphosate than wild type, and since MpMurA and MpEPSPS are structurally similar, we hypothesized that MpMurA confers glyphosate tolerance by catalyzing the same reaction as EPSPS—the conversion of S3P and PEP to EPSP and phosphate. To test this hypothesis, we measured the enzymatic activity of purified MpMurA enzyme with S3P and PEP as substrates (Fig. 5). We measured the amount of inorganic phosphate produced by MpMurA incubated with S3P and PEP as substrates (36, 37). As controls, the activity of purified MpEPSPS with the same substrates, and the activity of MpMurA with its canonical substrates, UDP-GlcNAc and PEP, were measured (Fig. 5*A*). Phosphate was produced by MpMurA in the presence of S3P and PEP, which is consistent with MpMurA catalyzing the same reaction as EPSPS. The rate of reaction of MpMurA was approximately 100-fold less than that of MpEPSPS. The reaction rate of MpMurA-catalyzed reaction of S3P and PEP was approximately 8-fold higher than MpMurA with the substrates UDP-GlcNAc and PEP. These data indicate that both MpMurA and MpEPSPS catalyze a reaction between S3P and PEP, producing inorganic phosphate. Conversely, neither MpEPSPS nor AtEPSPS catalyze the reaction between UDP-GlcNAc and PEP (*SI Appendix*, Fig. S6*B*). These data are consistent with MpMurA catalyzing the transfer of the enolpyruvyl moiety of PEP to both the 3’-hydroxyl of UDP-GlcNAc and S3P but MpEPSPS only to S3P.

To determine whether MpMurA catalyzes the production of EPSP from S3P and PEP substrates, liquid chromatography–tandem mass spectrometry (LC-MS/MS) was employed to detect the accumulation of EPSP (Fig. 5 *B* and *C*). To develop an LC-MS/MS method for the quantification of EPSP, we separated the reaction products of MpEPSPS, incubated with S3P and PEP, using hydrophilic interaction liquid chromatography (HILIC) directly coupled to mass spectrometry in the negative ion mode. We detected a peak at *m/z* 323.1, corresponding to the expected *m/z* value of EPSP. Upon fragmentation (Fig. 5*B*), we observed fragments at *m/z* 79 and *m/z* 97, characteristic of phosphorylated metabolites. The fragments at *m/z* 135 and *m/z* 137 likely correspond to EPSP having lost both the phosphate and the PEP-related structures upon fragmentation. By employing selected reaction monitoring (SRM), we then detected and quantified EPSP when MpEPSPS was incubated with S3P and PEP (Fig. 5*C*). EPSP was also detected and quantified when MpMurA was incubated with the same substrates. No EPSP was detected in the negative controls containing the enzyme alone or the substrates alone (chromatographs depicting the peaks corresponding to the substrates S3P and PEP can be found in *SI Appendix*, Fig. S6). The quantity of EPSP produced per mg of enzyme, measured in ion counts, was approximately 5,500-fold less for MpMurA (1.24 × 10^6^ ion counts/mg enzyme) than measured for MpEPSPS (6.82 × 10^9^ ion counts/mg enzyme) (Fig. 5*D*). This indicates that MpMurA catalyzes the reaction of S3P and PEP to form EPSP, although it is catalytically less active than MpEPSPS. Accumulation of EPSP product when MpMurA is incubated with S3P and PEP substrates demonstrates that MpMurA can catalyze the same reaction as MpEPSPS.

Glyphosate inhibits EPSPS activity by competing with PEP substrate for binding to the enzyme (28, 38). To test whether MpMurA was inhibited by glyphosate, the quantity of EPSP produced by MpMurA was measured at 8 concentrations of glyphosate using LC-MS/MS (Fig. 5*E*). The quantity of EPSP produced by MpEPSPS positive control enzyme decreased with increasing concentrations of glyphosate, consistent with the known inhibition of the forward reaction by the herbicide. The quantity of EPSP produced by MpMurA was also reduced by glyphosate, following a similar dose–response relationship as MpEPSPS. However, MpMurA was slightly more sensitive to glyphosate than MpEPSPS (Fig. 5*F*). Inhibition of MpMurA by glyphosate is consistent with both MpMurA and MpEPSPS catalyzing the production of EPSP and phosphate from S3P and PEP substrates.

Together, these data demonstrate that there are two enzymatic mechanisms that produce EPSP in *M. polymorpha*—one catalyzed by MpEPSPS and the other catalyzed by MpMurA.

## Discussion

We report the finding that MurA protein from the bryophyte *M. polymorpha* catalyzes the production of EPSP from S3P and PEP substrates, the same reaction catalyzed by the enzyme EPSPS, the glyphosate target. We also demonstrate that MurA is required for glyphosate tolerance *in M. polymorpha,* and heterologous expression of Mp*MurA* confers glyphosate resistance in the angiosperm *A. thaliana.* Since bryophytes encode a MurA enzyme and are glyphosate tolerant, and MurA is absent in most angiosperms which are generally glyphosate-sensitive, we propose that MurA-mediated glyphosate tolerance is a conserved ancestral trait among bryophytes. MurA activity could account for the anecdotal evidence of glyphosate-tolerant bryophytes reported in the horticultural media and literature (14–16).

Bacterial MurA catalyzes the transfer of the enolpyruvyl moiety from PEP to UDP-GlcNAc during peptidoglycan biosynthesis (39, 40). In plants that encode MurA, MurA catalytic activity is involved in the formation of the chloroplast wall and chloroplast division (18–20, 33). This is consistent with our finding that *M. polymorpha* Mp*murA* loss-of-function mutants have a macrochloroplast phenotype, and even mutations resulting in the change of few amino acids lead to a macrochloroplast phenotype. Here, we present a role of MurA in *M. polymorpha* which confers glyphosate tolerance. The finding that MpMurA catalyzes the transfer of the enolpyruvyl moiety from PEP to S3P forming the EPSP product demonstrates that MpMurA is involved in an independent route of EPSP synthesis in *M. polymorpha*. This is in addition to the canonical pathway catalyzed by EPSPS. The increased glyphosate sensitivity of Mp*murA* loss-of-function mutants, the higher glyphosate tolerance of Mp*MurA* overexpression lines in *M. polymorpha*, and the glyphosate resistance in *A. thaliana* expressing Mp*MurA* support the hypothesis that the catalysis of EPSP from PEP and S3P substrates by two different routes makes bryophytes glyphosate tolerant.

Despite demonstrating Mp*MurA* is required for glyphosate tolerance in *M. polymorpha* and Mp*MurA* expression confers resistance in *A. thaliana*, the mechanism of MurA-mediated glyphosate tolerance is not entirely clear, because MpMurA, like MpEPSPS, is sensitive glyphosate in vitro. Despite both being inhibited by glyphosate, it is possible that the presence of two enzymes—MpEPSPS and MpMurA—that synthesize EPSP, is sufficient to confer tolerance, while one gene is insufficient. There is evidence that the number of genes encoding EPSPS is positively correlated with glyphosate resistance. EPSPS gene amplification in wild populations is causally associated with glyphosate-resistant weeds such as *Amaranthus palmeri, Lolium multiflorum,* and *Kochia scoparia* (41–45). Such gene amplification was accompanied by higher steady state levels of EPSPS mRNA than in sensitive plants with no gene amplification, and this in turn results in higher EPSPS activity than in sensitive lines. However, we show that *MurA* expressed at lower levels in both angiosperms and *M. polymorpha* than *EPSPS*. Alternatively, the binding of glyphosate to MurA could contribute to resistance by decreasing the amount of the inhibitor that is available to bind to EPSPS. According to this model, if glyphosate binds to the active sites of MurA enzymes and EPSPS enzymes, a greater proportion of the EPSPS enzyme population would remain unbound and active than in the absence of MurA. This proportion of uninhibited EPSPS, and the fact that MurA could contribute to EPSP synthesis, could be sufficient to confer a degree of glyphosate tolerance in bryophytes. Consistent with this hypothesis, we show that MurA is sensitive to glyphosate, suggesting that glyphosate indeed binds to the enzyme. We also show that *murA* loss-of-function does not have the same effect on plant growth as glyphosate treatment. Therefore, although MurA is inhibited by glyphosate, which prevents the inhibition of EPSPS, this does not result to a reduction in plant growth.

While the presence of MurA is correlated with glyphosate tolerance across the major clades of land plants, this does not hold in every species. Some glyphosate-sensitive angiosperms encode MurA proteins. However, in two of these angiosperms, *S. tuberosum* and *N. attenuata*, expression of *MurA* is lower than *EPSPS* in all tissues (46, 47). Furthermore, in the leaves, onto which glyphosate is usually applied, there is 100-fold less *MurA* expression than *EPSPS* in *N. attenuata*, and no expression of *MurA* in *S. tuberosum* (46, 47). Therefore, in these glyphosate-sensitive angiosperm species that encode a *MurA* gene, the relative abundance of MurA enzyme may be insufficient to confer glyphosate tolerance and lack of *MurA* expression in some tissues but not others could result in sensitivity. In *M. polymorpha, MurA* is expressed throughout the plant indicating that MurA contributes to tolerance in every tissue (32). Tissue-specific sensitivity in angiosperms could make plants susceptible to glyphosate-induced death upon glyphosate exposure. Furthermore, there are subtle structural differences between the *M. polymorpha* MurA and that from *S. tuberosum*. Such differences could lead to a higher affinity for glyphosate in *M. polymorpha* MurA compared to *S. tuberosum* MurA, which could result in less EPSPS inhibition. Regardless, the observation that reducing levels of Mp*MurA* expression in loss-of-function *M. polymorpha* mutants makes plants glyphosate sensitive, and overexpressing Mp*MurA* in *M. polymorpha* makes plants more tolerant, demonstrates that Mp*MurA* is necessary and sufficient for glyphosate tolerance of bryophytes. Furthermore, heterologous expression of Mp*MurA* in *A. thaliana* confers glyphosate resistance, demonstrating that Mp*MurA* can confer glyphosate resistance when expressed in a glyphosate-sensitive plant.

MurA and EPSPS are closely related enzymes that together constitute a monophyletic clade of structurally similar enolpyruvyl transferases that utilize PEP as a substrate. We showed that both of these enzymes were likely derived from an ancestral enolpyruvyl transferase among the bacteria. Therefore, by parsimony, we hypothesize that the last common ancestor of EPSPS and MurA also catalyzed the production of EPSP from these substrates. If correct, this would suggest that MurA enzymes catalyze EPSP production in some bacteria, in addition to their canonical role in the first step of peptidoglycan synthesis where they catalyze the production of UDP-*N*-acetylglucosamine enolpyruvate (EP-UDP-GlcNAc) from PEP and UDP-*N*-acetylglucosamine (UDP-GlcNAc). However, bacterial MurA enzymes producing EPSP have not been reported in the literature. Therefore, we speculate that the production of EPSP by MurA enzymes is ancestral and has been conserved among most archaeplastida but lost from the bacterial lineage. The existence of both mechanisms of EPSP synthesis—catalyzed by EPSPS and MurA—results in glyphosate tolerance in *M. polymorpha* and perhaps all bryophytes.

## Materials and Methods

### Plant Lines and Growth Conditions.

Wild-type *M. polymorpha* lines were the laboratory accession Takaragaike-1 (Tak-1) and Takaragaike-2 (Tak-2) (48). The wild-type *A. thaliana* line used was Col-0. *L. cruciata* plants were collected from the wild. *P. patens* plants used were from the Gransden wild-type strain (49).

### Plant Glyphosate Dose–Response and Growth Assays.

To assess the glyphosate tolerance in the land *M. polymorpha*, *P. patens* and *L.cruciata* plants were grown for 14 d on solid ½ Gamborg media supplemented with varying glyphosate (Sigma-Aldrich, 3422) concentrations. All plant areas were normalized to the area of untreated plants of the same species. Other glyphosate and chlorsulfuron sensitivity assays were carried out by calculating the ratio between the area of treated plants and untreated control plants of the same genotype at a single herbicide concentration. The calculated ratio was used as a measure of herbicide sensitivity. Plant growth rates were assayed by imaging plant area each day between day 5 and 14 after plating gemmae. All plants were then imaged using a Berthold Nightowl II LB 983 in vivo imaging system (Berthold, Wildbad, Germany) following 120 s of white light exposure. The system detects chlorophyll autofluorescence at 560 nm, and the Berthold indoGO™ software package was used to determine the area of living autofluorescing tissue.

### Imaging of Plants and Chloroplasts.

Plants were imaged using a Keyence VHX7000 digital microscope equipped with VH-720R lens and VHX-7020 camera. Wild-type and mutant chloroplasts were imaged in 3-d-old gemmae using the Keyence VHX7000 digital microscope equipped with VH-700R/Z00T lenses and VHX-7020 camera.

### Expression and Purification MpEPSPS, MpMurA, and AtEPSPS Proteins.

The amino acid sequences, with the chloroplast transit peptides removed, of EPSPS proteins from *A. thaliana* (AT2G45300) and *M. polymorpha* (Mp6g04140.1), and MpMurA (Mp5g14110.1) from *M. polymorpha* were used for reverse transcription, codon optimization, and synthesis of DNA by Twist Bioscience. Expression constructs were transformed into *E. coli* strain BI21(DE3), cultured in liquid LB media with kanamycin at 37 °C overnight. Cultures were then diluted 1:250 in 2 l TB media with kanamycin and 1.5% w/v lactose and incubated with shaking (220 rpm) at 25 °C for 24 h. The cells were harvested by centrifugation at 5,313 rcf for 30 mins at 4 °C. The cell pellets were resuspended in 150 ml of Ni A buffer (50 mM Hepes (8.0), 300 mM NaCl, 20 mM Imidazole) supplemented with benzonase and protease inhibitors, followed by sonication (19 mm probe, 5 min; pulse on 1 s, pulse off 2 s, 60% amplitude). The intracellular soluble fraction was isolated by centrifugation at 41,656 rcf for 30 mins at 4 °C. His-tagged proteins were purified using Immobilized Metal Affinity Chromatography (IMAC) on 5 ml HisTrap FF columns, followed by His-tag removal with 3C protease. The tag-free proteins were purified from the 3C protease and the remaining tagged protein via Reverse IMAC. Samples were concentrated to 1.5 ml using vivaspin 20 (MWCO 10 kDa) and subjected to Size Exclusion Chromatography on a HiLoad 16/60 Superdex200 pg column equilibrated with SEC buffer (50 mM Hepes pH 8.0, 150 mM NaCl, 1 mM EDTA, 1 mM TCEP, 5% glycerol w/v) at a flow rate of 1 ml/min.

### Measurement of Glyphosate, Shikimate and Shikimate-3-Phosphate in Plant Tissue Using LC-MS/MS.

*M. polymorpha* and *A. thaliana* plants were grown for a total of 14 d on cellophane and transferred to media supplemented with 5 μM of glyphosate for 2 d. 150 mg of plant tissue was placed in 2 ml Eppendorf tubes containing a sterile metal bead. Six biological replicates were collected for each treatment group. Samples were frozen in liquid nitrogen and the tissue ground into a powder for 2 mins using a tissuelyser (Retsch MM400). Subsequent steps were conducted as close to 4 °C as possible. To each sample, 500 μl of methanol:acetonitrile:H_2_O (2:2:1, v/v, cooled to −20 °C) was added, and the mixture homogenized for 2 mins in the tissuelyser. Samples were incubated at −20 °C for 1 h and centrifuged for 2 mins at 14,000 rpm. Supernatants were collected and transferred to new Eppendorf tubes and stored at −20 °C. To the pellet, 400 μl of 80% methanol:water (v/v, cooled to −20 °C) was added, followed by vertexing for 1 min. Samples were incubated at −20 °C for 1 h and centrifuged for 2 mins at 14,000 rpm. Supernatants from the first extraction were combined with those from the second extraction for each sample, and samples were incubated for 2 h at −20 °C. Samples were then centrifuged for 10 mins at 14,000 rpm. 800 μl of supernatant was transferred to new Eppendorf tubes, flash frozen in liquid nitrogen, and stored at −80 °C until analysis.

Metabolite extracts from *M. polymorpha* and *A. thaliana* plants were analyzed by HILIC, directly coupled with LC-MS/MS as for the detection EPSP. The following SRM transitions were employed for quantification in the negative ion mode: *m/z* 168 to *m/z* 150 (glyphosate), *m/z* 173 to *m/z* 93 (shikimate), *m/z* 253 to *m/z* 97 (shikimate-3-phosphate). Authentic standards were used for determining optimal collision energies of the SRM transitions and for validating experimental retention times via standard addition to a pooled quality control sample.

### Protein Structure Prediction Using AlphaFold.

MpEPSPS and MpMurA protein structures from *M. polymorpha* were predicted using AlphaFold (50). Amino acid sequences of MpEPSPS (Mp6g04140.1) and MpMurA (Mp5g14110.1) were used as inputs. ChimeraX (51) was employed to compare the resulting structures with the chloroplast transit peptides removed.

### Phylogenetic Analysis.

EPSPS and MurA amino acid sequences were identified through a protein BLASTp using MpEPSPS (Mp6g04140.1) and MpMurA (Mp5g14110.1) as queries against reference proteomes of archaeplastida, bacteria, and archaea species. Inclusion criterion was an E-value of less than 1E-10, and confirmation as EPSPS or MurA proteins was insured through an InterPro domain search (52). Confirmed EPSPS and MurA protein sequences were aligned in MAFFT using the L-INS-I algorithm (53) and visualized in Unipro Ugene v. 45.1 (54). Sequences were manually trimmed to retain conserved regions while removing large nonconserved regions and gaps. The final trimmed amino acid sequence alignment was subjected to maximum-likelihood phylogenetic analysis using PhyML 3.0 with an estimated gamma distribution parameter and the WAG + G+I model of amino acid substitution (55). Support values were generated using a Chi^2^-based approximate likelihood ratio test (aLRT). Resulting phylogenies were visualized in FigTree v1.4.4 (56), followed by annotation in Inkscape v1.0.2. The presence/absence of EPSPS and MurA was plotted onto a cladogram with a supported topology of archaeplastida species based on: (29–31).

### Overexpression Construct Making.

To make the construct *35S:*Mp*MurA*, the DNA sequence of *M. polymorpha MurA* was synthesized by Twist Bioscience with the correct overhangs and BsaI sites removed. Synthesized products were then cloned using GreenGate cloning (57). Products were cloned into entry module pGGI000, transformed into DH5-α *E. coli* cells, and purified using the Vienna Biocenter’s in-house kit. Subsequently, this plasmid, the promotor module (35S), and the terminator module (RBCS) were combined with the destination vector (pGGZ003). The same process was carried out for constructs carrying YFP, although the YFP module was available in the GreenGate kit.

### Design and Cloning of CRISPR-Cas9 Vectors.

*M. polymorpha MurA* (Mp5g14110.1) and *MurF* (Mp1g21490) genes were mutagenized using CRISPR-Cas9. Guide RNAs targeting specific gene regions with “NGG” sites (PAM sequence) were designed in the 5’ direction (58) (*SI Appendix*, Figs. S4 and S5). The CRISPR-Cas9 vectors were then assembled using the OpenPlant toolkit which utilizes GoldenGate cloning methods (59).

### *Agrobacterium tumefaciens*-Mediated Transformation of *M. polymorpha* Sporelings.

Vectors were introduced into *M. polymorpha* sporelings generated from a cross between Takaragaike-1 (Tak-1) and Takaragaike-2 (Tak-2) plants using a modified method from ref. 48. *A. tumefaciens* strain GV3101 was transformed with vectors via electroporation. This strain is resistant to rifampicin and gentamycin, and successful transformants were hygromycin resistant. Transformed *A. tumefaciens* were cultured on solid LB media with 50 μg ml^−1^ hygromycin, gentamycin, and rifampicin for 2 d at 28 °C to select colonies expressing the vectors. Single colonies were cultivated in 5 ml liquid LB media supplemented with hygromycin, gentamycin, and rifampicin, then induced with 100 μM acetosyringone in M51C media (48, 60) for 3 to 6 h at 28 °C, shaking at 150 rpm. 1 ml of the induced culture was combined with a 25 ml culture of *M. polymorpha* sporelings (resulting from a cross between Tak-1 and Tak-2 accessions) grown for 5 d in M51C media at 23 °C under 50 to 60 μmol m^−2^ s^−1^ white light. The sporeling and *A. tumefaciens* culture was cocultivated with 100 μM acetosyringone for 2 d at 23 °C, shaking at 120 rpm. The culture was then filtered through a 50 μM cell strainer to collect the sporelings and *A. tumefaciens* was removed by washing with 150 ml dH2O. Sporelings were plated on solid ½ Gamborg medium with 1% agar, supplemented with 100 μg ml^−1^ cefotaxime (to eliminate any remaining *A. tumefaciens*) and 10 μg ml^−1^ hygromycin for selection. The selection occurred at 23 °C under 10 to 30 μmol m^−2^ s^−1^ white light for 1 to 2 wk. Surviving plants were then transferred to new plates with solid ½ Gamborg medium with 1% agar, supplemented with 100 μg ml^−1^ cefotaxime and 10 μg ml^−1^ hygromycin, for an additional week.

### *A. tumefaciens*-Mediated Transformation of *A. thaliana* Plants.

Single colonies of *A. tumefaciens* transformed with the overexpression vectors were grown in 5 ml of liquid LB media supplemented with hygromycin, gentamycin, and rifampicin, and incubated for 2 d with shaking at 150 rpm at 28 °C. The culture was then used to inoculate 100 ml of liquid LB with the same antibiotics and incubated for a further 2 d. The 100 ml culture was centrifuged, and resulting pellet washed with 20 ml of a 5% sucrose water solution. The pellet was resuspended in 50 ml of 5% sucrose water solution, with the addition of 10 μl of SILWET L-77. Healthy *A. thaliana* Col-0 plants had their flowers submerged in the *A. tumefaciens* solution for 5 mins in a beaker. Plants were then placed in the dark overnight. Seeds from these plants were plated on solid MS media (2.2 g/l MS salt, 0.5 g/l MES, 0.8% agar) supplemented with 10 μg ml^−1^ hygromycin. The seeds were stratified for 2 d at 4 °C and in the dark before germinating in a chamber with 16 h of 85 μmol m^−2^ s^−1^ white light and 8 h of darkness, growing in these conditions for 2 wk. Selected growing plants were transferred to soil, and homozygous lines for the overexpression constructs were generated.

### *MurA* Expression Quantification Using RT-qPCR.

Plants were grown for 2-weeks, and total RNA was extracted using RNeasy plant mini kits (Qiagen) according to the manufacturer’s instructions. DNA was removed using RNase-free DNase set (Qiagen) following the manufacturer’s instructions. RNA quantities were measured using an Implen N60 nanophotometer. 1 μg of RNA was converted to cDNA using the LunaScript RT SuperMix kit (NEB) in a 20 μl reaction. Primers for the Mp*MurA* gene and the _pro_*35S:*Mp*MurA* construct were designed using the Eurofins primer design tool (https://eurofinsgenomics.eu/en/ecom/tools/pcr-primer-design). For *M. polymorpha* plants, Mp*APT* and Mp*ACT* were used as reference genes (61). Reference genes for *A. thaliana* plants were At*UBC21* and At*ACT2*. The list of primers used for RT-qPCR can be found in *SI Appendix*, Table S1.

RT-qPCR experiments were performed in triplicate in either 384- or 96-well format. In the 384-well format, 10 μl reaction mixtures contained 5 μl Luna Universal qPCR master mix, 0.25 μl forward primer, 0.25 μl reverse primer (both 10 μM), 1 μl of 1:2 diluted cDNA and 3.5 μl nuclease free water. These quantities were all doubled in the 96-well format. The RT-qPCR was run as follows on a QuantStudio 7 cycler (Applied Biosystems) (384-well) or a LightCycler 96 (Roche) (96-well) machine: step 1: 95 °C for 60 s, step 2: 95 °C for 15 s, step 3: 60 °C for 30 s (plus plate read): step 4: melt curve of 60 to 95 °C. Steps 2 to 3 were repeated 40 times. Relative expression of *MurA* in loss- and gain-of-function mutants was calculated as 2-^ΔCq^ comparing the mean Cq values of each line to the reference sample mean. Relative expression of *MurA* and *EPSPS* in Tak-1 and Tak-2 was calculated using a modified 2^−(ΔΔCq)^ that accounts for differences in primer efficiencies, and incorporates multiple control genes (Mp*ACTIN7* and Mp*APT*) (62, 63).

### AtEPSPS, MpEPSPS, and MpMurA Activity Assays and Detection of EPSP with LC-MS/MS.

Purified MpEPSPS and MpMurA enzyme activities and kinetics of MpEPSPS were assayed following an adapted protocol based on: (36, 64). Enzymatic activities were determined by measuring inorganic phosphate production in the forward reaction using S3P (TRC, S357026) or UDP-GlcNAc (Sigma-Aldrich, U4375) and PEP (Sigma-Aldrich, 10108294001) as substrates in 96-well plates. The quantification relied on a malachite green phosphate assay kit (Sigma-Aldrich, MAK307), and absorbance changes at 650 nm were measured using a BioTek Synergy H1 plate reader. Enzyme activity was expressed as Units mg^−1^ (U mg^−1^), representing micromoles of phosphate produced, per minute, per milligram of enzyme. Each 80 μl reaction mixture contained 50 mM HEPES (pH 7.5), 100 mM KCl, 2 mM DTT, and 1 mM of each substrate. Enzymatic reactions were initiated with enzyme addition and incubated for 30 mins at 25 °C, followed by halting the reactions with malachite green and allowing for 30 mins of color development.

Enzyme activity was also determined by detection and measurement of the reaction product EPSP. Samples were analyzed by liquid chromatography directly coupled to hydrophilic interaction liquid chromatography (HILIC) mass spectrometry (LC-MS/MS). Following the addition of 60 μl acetonitrile to 40 μl sample and centrifugation, 1 μl of the supernatant was directly injected onto a polymeric iHILIC-(P) Classic HPLC column (HILICON, 100 × 2.1 mm; 5 μm) and the respective guard column, operated at a flow rate of 100 μl/min. A linear gradient (A: acetonitrile; B: 10 mM aqueous ammonium bicarbonate, supplemented with 0.1 μg/ml medronic acid) starting with 25% B and ramping up to 90% B in 14 mins has been used for separation. Using the selected reaction monitoring (SRM) mode of a TSQ Quantiva mass spectrometer (Thermo Fisher Scientific), the following SRM transitions were employed for EPSP quantification in the negative ion mode: *m/z* 167 to *m/z* 79 (PEP), *m/z* 253 to *m/z* 97 (S3P), *m/z* 323 to *m/z* 97 (EPSP; qualifier). Authentic standards of S3P and PEP were used for determining optimal collision energies and SRM transitions and for validating experimental retention times via standard addition. The data interpretation was performed using TraceFinder (Thermo Fisher Scientific).

## Supplementary Material

Appendix 01 (PDF)

Dataset S01 (TXT)

Dataset S02 (TXT)

## Data Availability

All study data are included in the article and/or supporting information.
