# Supplementary material for: MurA-catalyzed synthesis of 5-enolpyruvylshikimate-3-phosphate confers glyphosate tolerance in bryophytes
Source: Proc Natl Acad Sci U S A. 2024 Nov 11;121(47):e2412997121. doi: 10.1073/pnas.2412997121 (PMC11588093; doi:10.1073/pnas.2412997121)
Supplement: Supplementary file 1 — Appendix 01 (PDF) [file pnas.2412997121.sapp.pdf]

Supporting Information for
MurA-catalyzed synthesis of 5-enolpyruvylshikimate-3-phosphate confers glyphosate tolerance in bryophytes

Samuel Caygill, Thomas Köcher, Liam Dolan

Liam Dolan
Email: liam.dolan@gmi.oeaw.ac.at

This PDF file includes:

Figures S1 to S6
Tables S1
SI References

Other supporting materials for this manuscript include the following:

Datasets S1 and S2

Supporting Information Text

Plant Growth Conditions

All plants were grown on solid ½ Gamborg medium (1.5 g/l B5 Gamborg, 0.5 g/l MES hydrate, 1% sucrose, pH adjusted to 5.5) with 0.8% agar. Bryophyte plant lines were maintained through asexual propagation of gemmae or thallus or filament excision and grown in a growth chamber at 23 ℃ under 24-hour 50-60 μmol m^{-2} s^{-1} white light or at 20 ℃ under 16-hour 50-60 μmol m^{-2} s^{-1} white light supplemented with 30-40 μmol m^{-2} s^{-1} far-red light. *A. thaliana* plants were grown under a 16-hour day of 85 μmol m^{-2} s^{-1}.

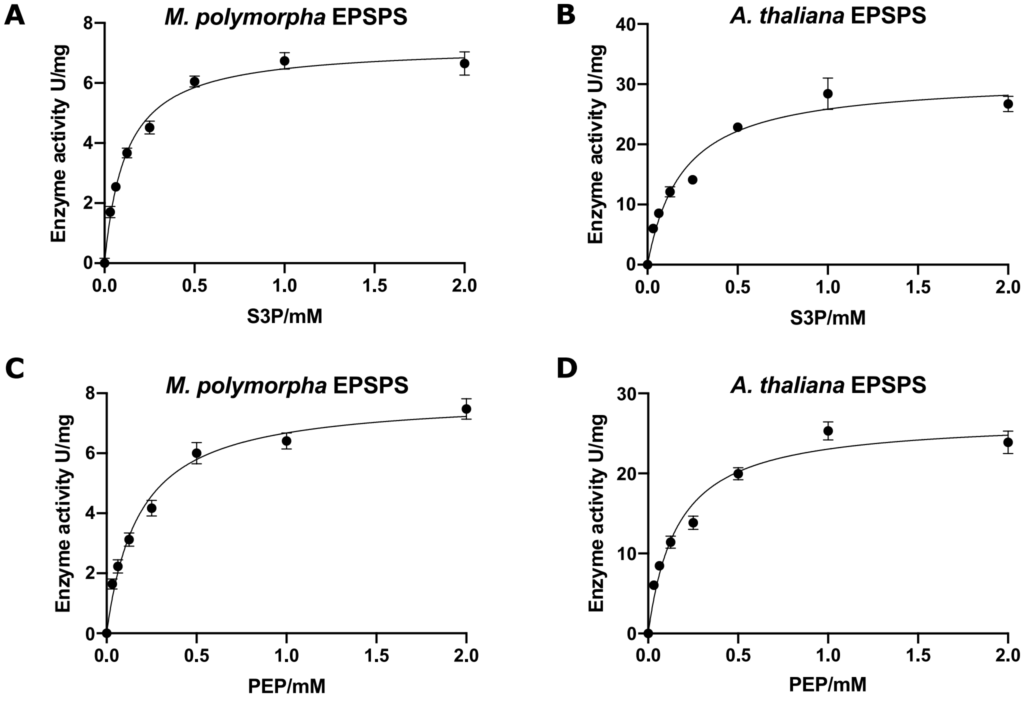

E

	K_m (PEP)	V_{max} (PEP)	K_{cat} (PEP)	K_{cat}/K_m (PEP)	K_m (S3P)	V_{max} (S3P)	K_{cat} (S3P)	K_{cat}/K_m (S3P)	IC_{50}
	mM	U mg^{-1}	s^{-1}	M^{-1}s^{-1}	mM	U mg^{-1}	min^{-1}	s^{-1}	mM
M. polymorpha EPSPS	0.1803 (0.02558)	7.880 (0.3271)	0.2522 (0.01047)	1.399x10^3 (2.0677x10^2)	0.1197 (0.01282)	7.248 (0.2050)	0.2320 (0.006561)	1.938x10^3 (2.146x10^2)	9.566 (9.123)
A. thaliana EPSPS	0.1636 (0.03375)	26.81 (1.579)	1.716 (0.1011)	1.0489x10^4 (2.225x10^3)	0.1967 (0.04463)	30.94 (2.100)	1.980 (0.1344)	1.0066x10^4 (2.384x10^3)	14.49 (5.983)

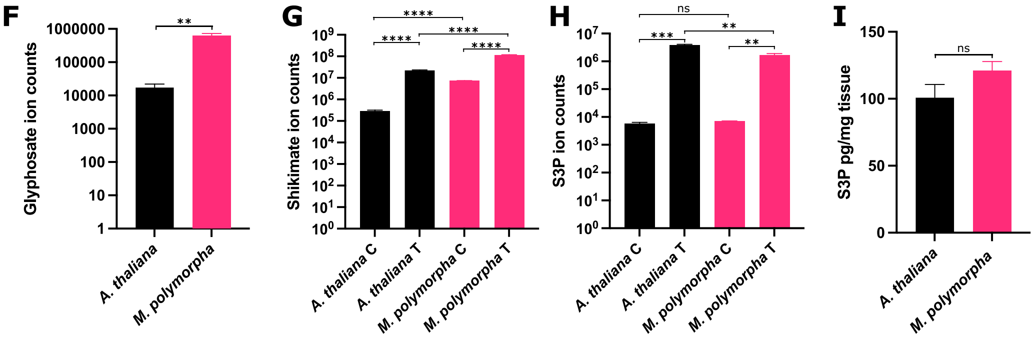

Fig. S1. Enzyme kinetics of *M. polymorpha* and *A. thaliana* EPSPS enzymes and glyphosate, S3P and shikimate levels in plant tissues

(A) Michaelis-Menten curve of *M. polymorpha* EPSPS for S3P substrate. **(B)** Michaelis-Menten curve of *A. thaliana* EPSPS for S3P substrate. **(C)** Michaelis-Menten curve of *M. polymorpha* EPSPS for PEP substrate. **(D)** Michaelis-Menten curve of *A. thaliana* EPSPS for PEP substrate. **(E)** Table summarising the kinetic parameters of *M. polymorpha* and *A. thaliana* EPSPS proteins. Standard errors are in parantheses. **(F)** Glyphosate quantity in ion counts in the thalli and leaves of *M. polymorpha* and *A. thaliana* respectively following 2 days of glyphosate treatment. **(G)** Shikimate quantity in ion counts in the thalli and leaves of *M. polymorpha* and *A. thaliana* respectively following 2 days of glyphosate treatment or in control plants. These data were used to calculate shikimate fold changes in Fig. 1 E. **(H)** S3P quantity in ion counts in the thalli and leaves of *M. polymorpha* and *A. thaliana* respectively following 2 days of glyphosate treatment or in control plants. These data were used to calculate S3P fold changes in Fig. 1 F. **(I)** Absolute quantity of S3P in control *M. polymorpha* and *A. thaliana* plants.

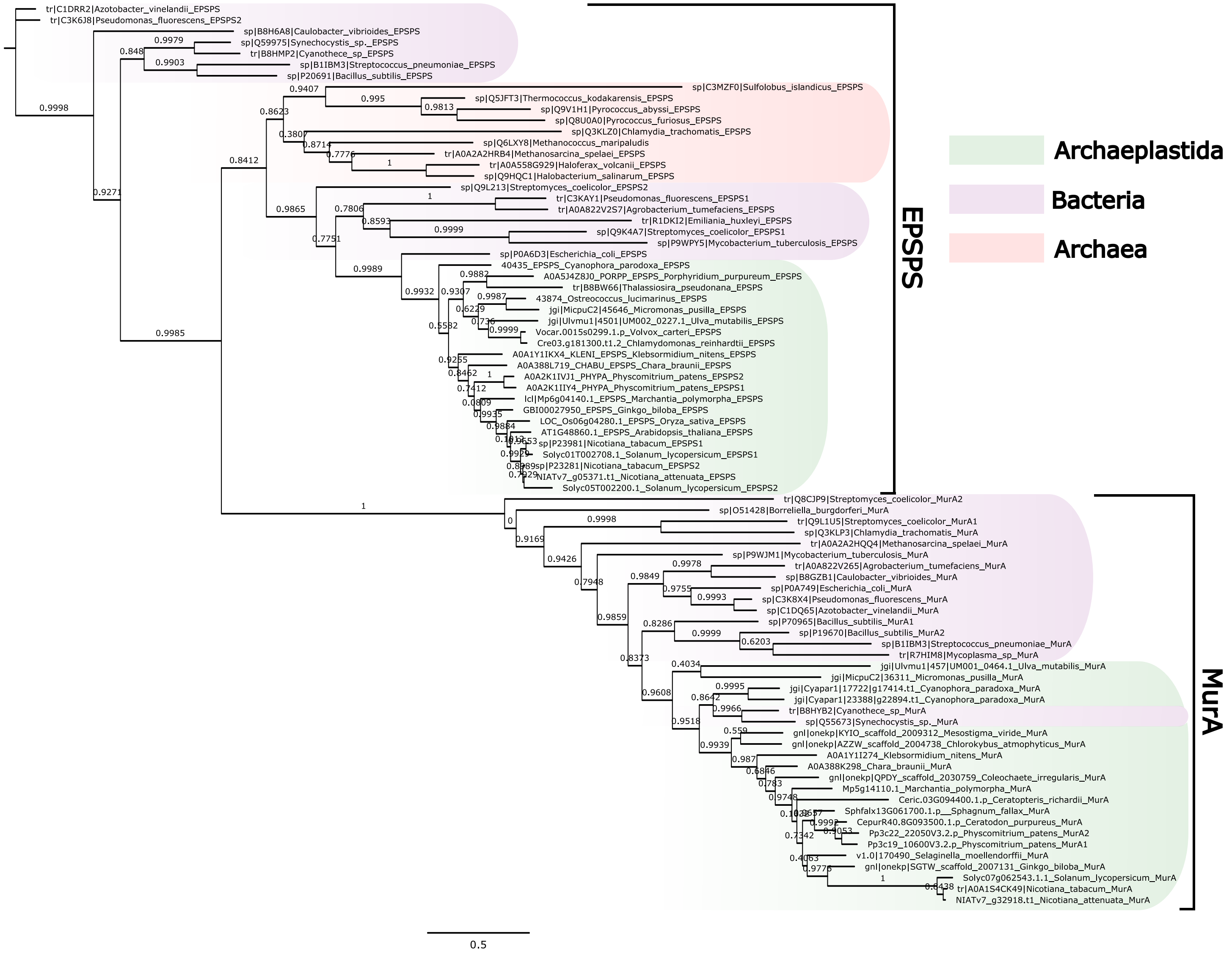

Fig. S2. MurA proteins evolved from an EPSPS-like protein in the bacterial lineage
Unrooted maximum likelihood tree of MurA amino acid sequences from Archaeplastida, bacteria and archaea species. Protein sequences were aligned using MAFFT with the L-INS-i algorithm. The phylogenetic tree was constructed using PhyML 3.0 [1] using an estimated gamma distribution model and the WAG+G+I model of amino acid substitution. Support values presented are from a Chi^2-based aLRT test.

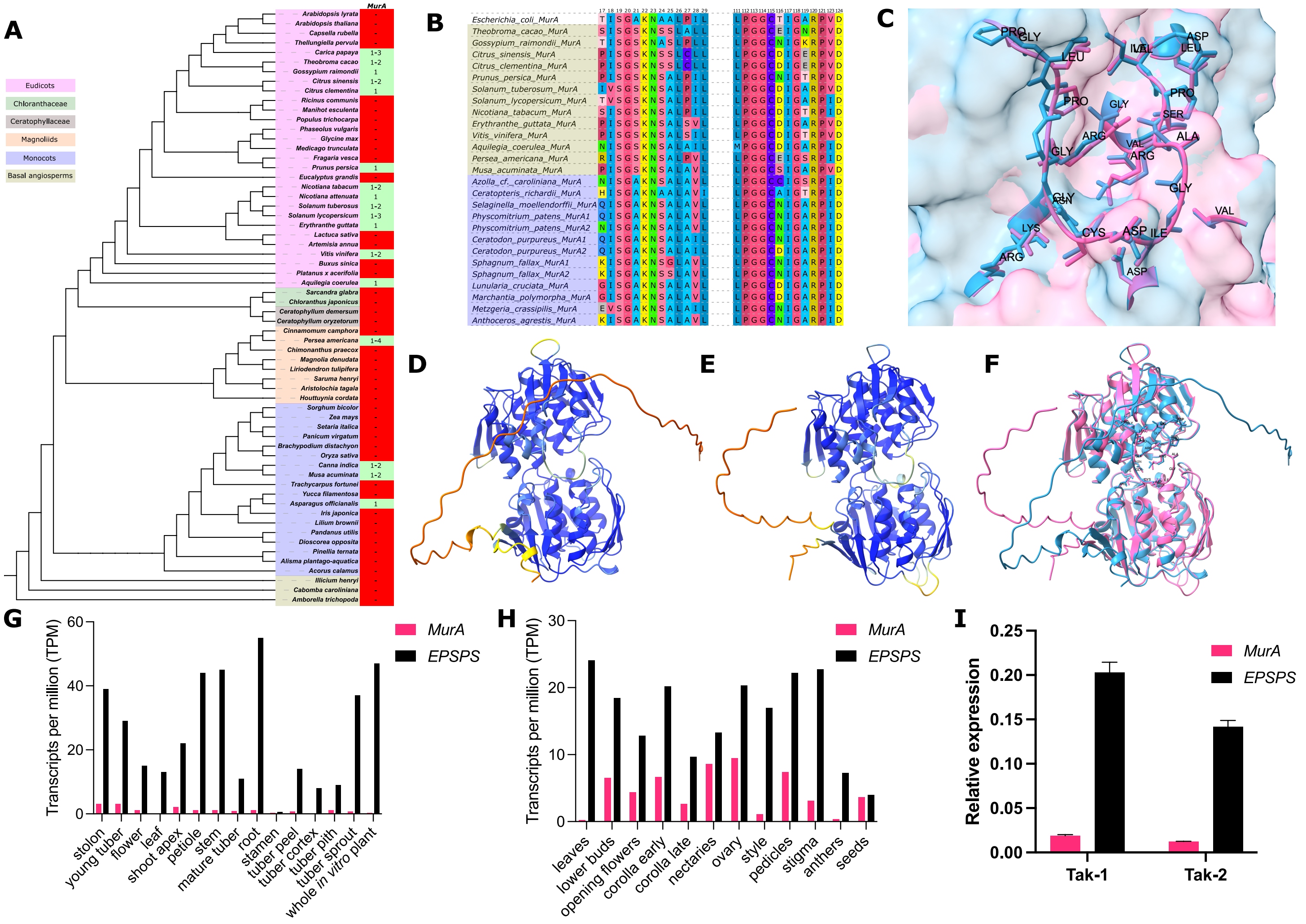

Fig. S3. Few angiosperms encode MurA and angiosperm MurAs are not expressed in leaves and are subtly different from non-angiosperm streptophyte MurAs

(A) Species tree based on [2–7]. Presence or absence of EPSPS and MurA in each species was determined using BLASTp searches with the amino acid sequence of each enzyme from *M. polymorpha* as a query. Green depicts presence, red absence, and the number of enzyme paralogs when present is given. **(B)** Multiple amino acid sequence alignments of angiosperm (green) and non-angiosperm streptophyte (blue) MurAs surrounding the residues Lys22 and Cys115 which are essential for catalysis. The MurA sequence from *E. coli* was also included as a reference. These residues are conserved however some differences in the surrounding residues can be seen. **(C)** Structural comparison of AlphaFold predictions of *Solanum tuberosus* (pink) and *M. polymorpha* (blue) of the region surrounding Cys115. **(D)** AlphaFold prediction of the MurA enzyme from *M. polymorpha*. **(E)** AlphaFold prediction of the MurA enzyme from *S. tuberosus*. **(F)** Overlay of the MurA enzymes from *M. polymorpha* (blue) and *S. tuberosus* (pink) produced in USCF ChimeraX using the matchmaker function. **(G)** Expression of *MurA* and *EPSPS* in *S. tuberosus* in different tissues adapted from [8]. **(H)** Expression of *MurA* and *EPSPS* in *Nicotiana attenuate* in different tissues adaoted from [9]. **(I)** Relative expression of Mp*MurA* in *M. polymorpha* Tak-1 and Tak-2 accessions calculated from fold-differences in mRNA abundance relative to two control genes (Mp*ACTIN7* and Mp*APT*).

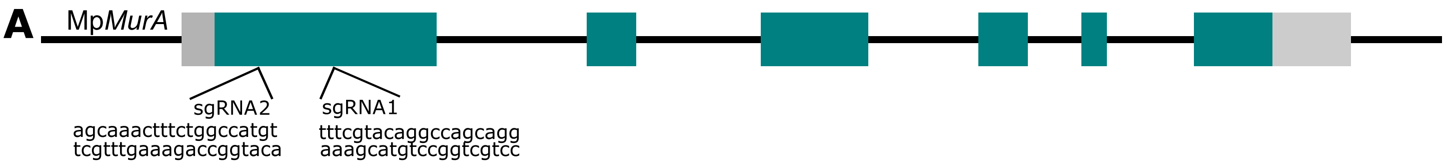

B

Plant line	Gene Sequence	Genetic Sequence change	Predicted protein sequence	Predicted change to translated protein
**sgRNA1**				
Wild-type	agcaaactttctggccatgtcggaataagt		SKLSGHVGIS	
Mp*murA*-10	agcaaactttc------tgtcggaataagt	6 bp deletion	SKLS--VGIS	Deletion of Gly, His
Mp*murA*-13	agcaaactttc------tgtcggaataagt	6 bp deletion	SKLS--VGIS	Deletion of Gly, His
Mp*murA*-17	agcaaactttctggccaagaactttctgtcggaataagt	9 bp insertion	SKLSGQELSVGIS	His > Gln substitution, insertion of Glu, Leu, Ser
**sgRNA2**				
Wild-type	cgtcctcctgctggcctgtacgaaattcg		ASSCWPVRNS	
Mp*murA*-8	gcgtcct-----------gtacgaaattcg	11 bp deletion	ASCTKF...*	Truncation 24 aa downstream
Mp*murA*-15	gcgtcctcc----------tacgaaattcg	10 bp deletion	ASSYEI...*	Truncation 9 aa downstream
Mp*murA*-23	gcgtcctcc---tggcctgtacgaaattcg	3 bp deletion	ASS-WPVRNS	Deletion of Cys

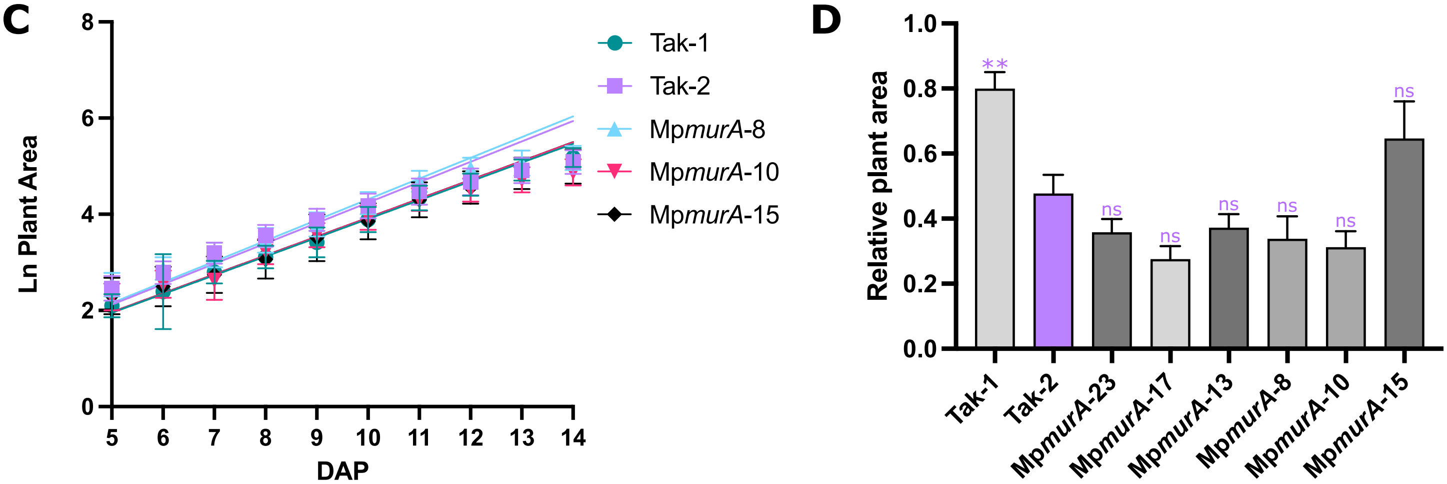

Fig. S4. Generation of predicted loss-of-function mutation in Mp*MurA* by CRISPR/Cas9 mutagenesis and chlorsulfuron sensitivity assay

(A) Mp*murA* mutant lines were generated using the CRISPR-Cas9 mutagenesis system with two guide RNAs designed to target the first exon of the Mp*MurA* gene carried by a vector containing the Cas9 gene which were transformed into *M. polymorpha* spores. **(B)** A summary of the nature of the mutations induced in Mp*MurA* induced by CRISPR-Cas9 mutagenesis. **(C)** Growth rates of *M. polymorpha* wild-type and Mp*murA* loss-of-function lines between 5 and 14 days after propagation (DAP) of gemmae. Plant areas were transformed by Ln and a simple linear regression fitted to determine growth rate (slope). There was no significant difference between the growth rates of all lines (one-way ANOVA, $p > 0.05$). **(D)** Plant area ratios of *M. polymorpha* wild-type and Mp*murA* loss-of-function lines grown on 0 nM and 20 nM of chlorsulfuron for 14 days. The auto-fluorescing areas were measured and the ratios between the treated and untreated plants calculated and plotted. Error bars are ±SE.

A

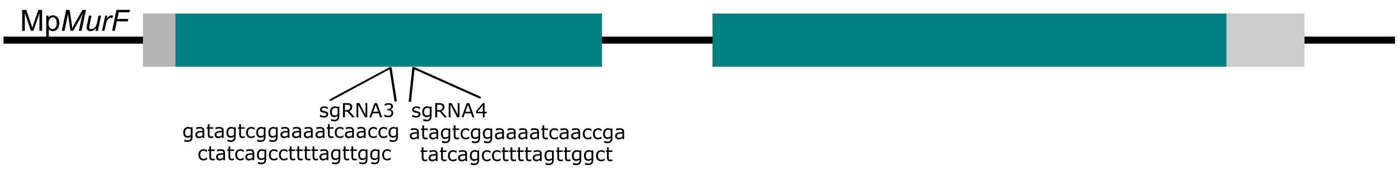

B

Plant line	Gene Sequence	Genetic Sequence change	Predicted protein sequence	Predicted change to translated protein
**sgRNA3 and sgRNA4**				
Wild-type	tgcaccgatagtcggaaaatcaaccgaggg		CTDSRKINRG	
Mp*murF*-1	tgcaccgat-------------------gg	19 bp deletion	CTDGNGFLLL*	Truncation 7 aa downstream
Mp*murF*-9	tgc-------------------ccgaggg	19 bp deletion	CTEGNGFLLL*	Truncation 8 aa downstream
Mp*murF*-12	tgcaccgatagtcggaaaaccattgccgaggg	2 bp insertion, 2 bp substitution	CTDSRKTIAE…*	Truncation 11 aa downstream
Mp*murF*-2	tgcaccgatagtcggaaaatcaa-cgaggg	1 bp deletion	CTDSRKINEG…*	Truncation 10 aa downstream
Mp*murF*-7	tgcaccgatagtcggaacggaattcgaggg	2 bp insertion, bp substitution	CTDSRNGITE…*	Truncation 12 aa downstream

C

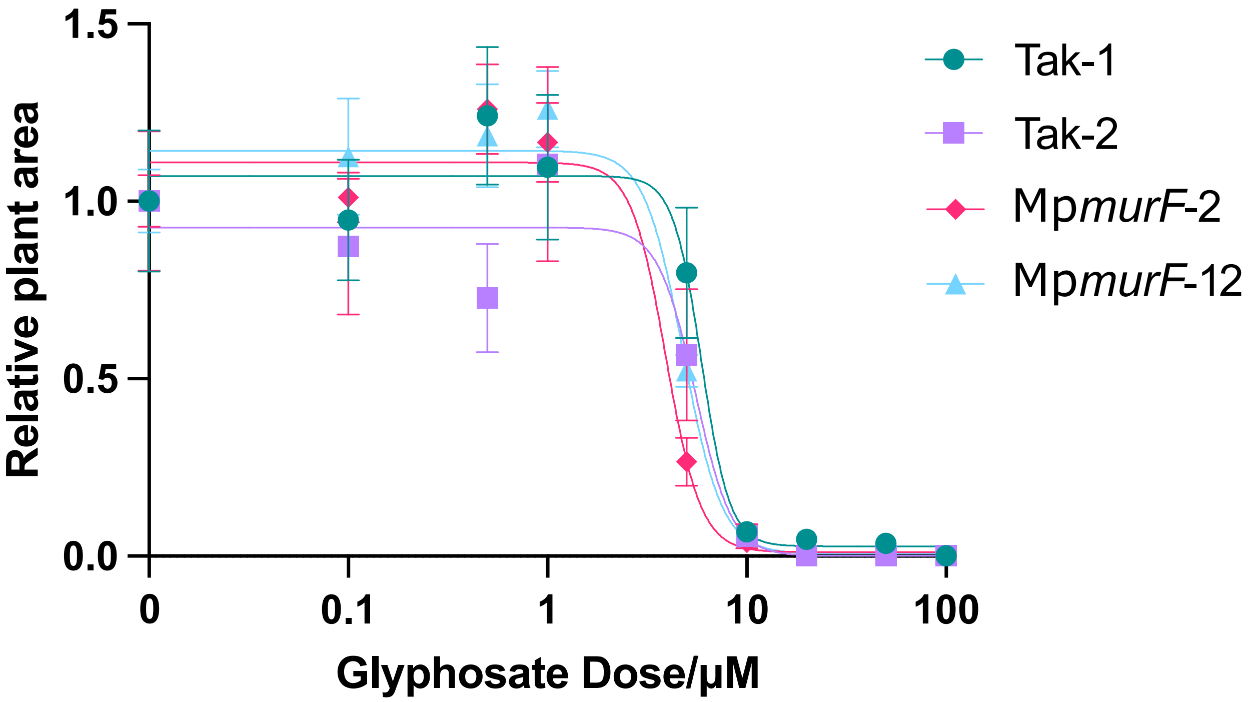

Fig. S5. Generation of predicted loss-of-function mutation in Mp*MurF* by CRISPR/Cas9 mutagenesis and dose-response assay to glyphosate for 2 putative mutants
(A) Mp*murF* mutant lines were generated using the CRISPR-Cas9 mutagenesis system with two guide RNAs designed to target the first exon of the Mp*MurF* gene carried by a vector containing the Cas9 gene which were transformed into *M. polymorpha* spores. **(B)** A summary of the mutations induced in Mp*MurF* induced by CRISPR-Cas9 mutagenesis. **(C)** Dose response curves of the plant areas of *M. polymorpha* wild-type and Mp*murF* loss-of-function plants grown for 14 days on glyphosate treated plates. The data are fitted with four-parameter log-logistic regression curves. Error bars are ±SE.

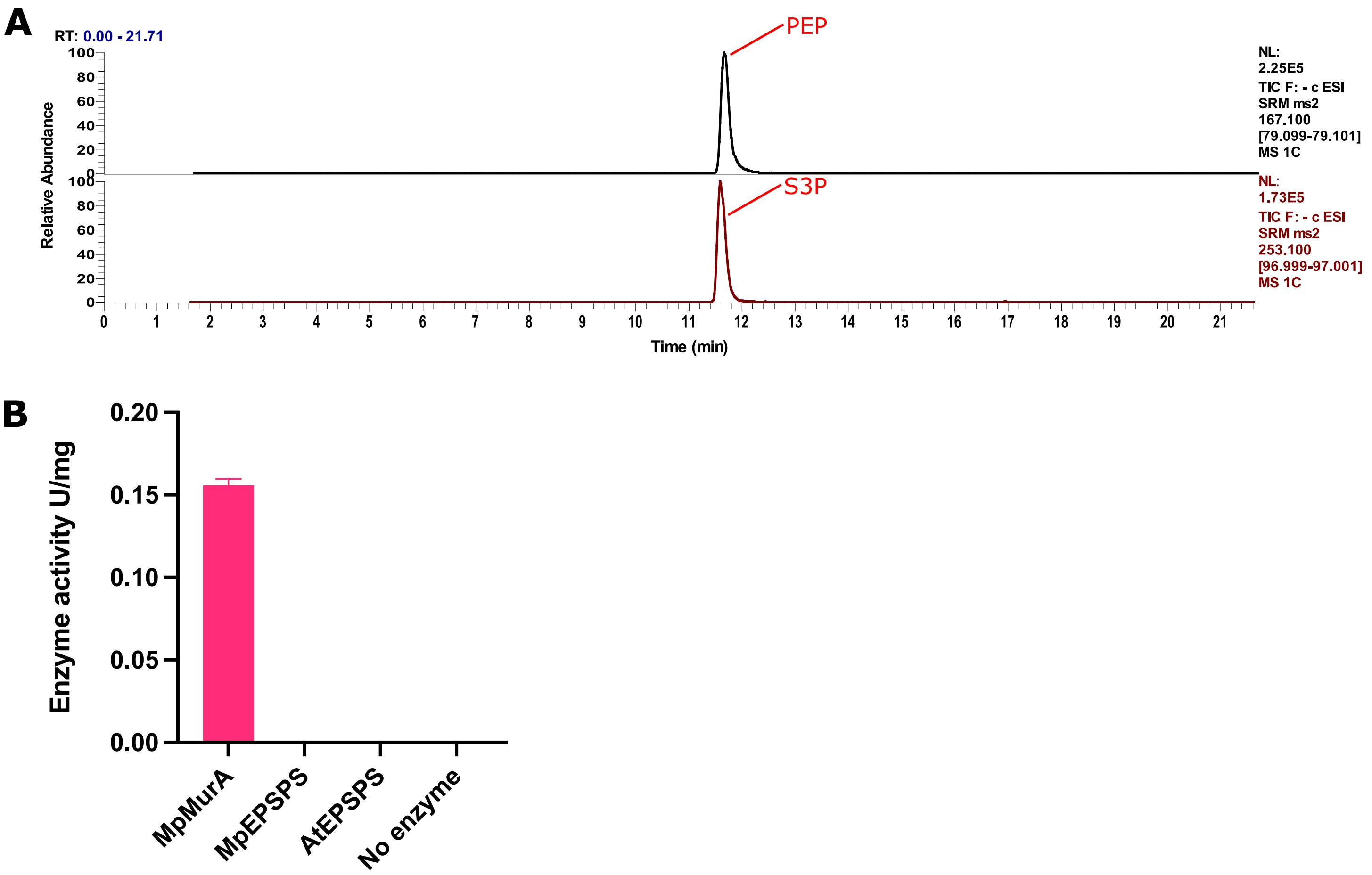

Figure S6. SRM chromatograms and activity of EPSPS enzymes with MurA substrates
(A) SRM chromatograms of S3P and PEP. **(B)** Enzymatic activity of MpMurA, MpEPSPS and AtEPSPS with the substrates of MurA, UDP-GlcNAc and PEP, measured as μmol phosphate produced/min of reaction time/mg of enzyme (U/mg).

	Sequence	Use
Mp-U6-seqF	GAGTTCGGATTGCTCTCTTTC	Sanger sequencing of CRISPR expression vector
murA1_fwd	ACCGAGCTCTTGGATCTCAGC	Genotyping Mp*murA* lines (sgRNA1, sgRNA2); sequencing Mp*murA* lines (sgRNA1, sgRNA2);
murA1_rev	ACGCAGAGTTAGCAGCGCTCG	Genotyping Mp*murA* lines (sgRNA1, sgRNA2);
murF1_fwd	TCTACCGATCGTTCACATGC	Genotyping Mp*murF* lines (sgRNA3, sgRNA4); sequencing Mp*murF* lines (sgRNA3, sgRNA4
murF1_rev	CTCAAAGTTCTCCATATGCG	Genotyping Mp*murF* lines (sgRNA3, sgRNA4)
murA3_fwd	GTGGAGATAAGGCAAGACAAG	qPCR of endogenous *M. polymorpha MurA*
murA3_rev	CTCCTGAGCCACATTTGAC	qPCR of endogenous *M. polymorpha MurA*
OEmurA2_fwd	ATCACTACGCTCCCTTACCC	qPCR of overexpression construct for *M. polymorpha* MurA
OEmurA2_rev	ATTTACCACAACCTTCGCAC	qPCR of overexpression construct for *M. polymorpha* MurA
epsps2_fwd	AGAGACAGAAAGGATGGTGG	qPCR of endogenous *M. polymorpha EPSPS*
epsps2_rev	CACAAGCAGCAAGAGAGAAG	qPCR of endogenous *M. polymorpha EPSPS*
MpACT7_fwd	AGGCATCTGGTATCCACGAG	qPCR of endogenous *M. polymorpha ACTIN* house-keeping gene
MpACT7_rev	ACATGGTCGTTCCTCCAGAC	qPCR of endogenous *M. polymorpha ACTIN* house-keeping gene
MpAPT3_fwd	CGAAAGCCCAAGAAGCTACC	qPCR of endogenous *M. polymorpha APT* house-keeping gene
MpAPT3_rev	GTACCCCCGGTTGCAATAAG	qPCR of endogenous *M. polymorpha APT* house-keeping gene
AtACT2_fwd	GCCATCCAAGCTGTTCTCTC	qPCR of endogenous *A. thaliana ACTIN2* house-keeping gene
AtACT2_rev	ACCCTCGTAGATTGGCACAG	qPCR of endogenous *A. thaliana ACTIN2* house-keeping gene
AtUBC21_fwd	TCAAATGGACCGCTCTTATC	qPCR of endogenous *A. thaliana UBC21* house-keeping gene
AtUBC21_rev	CACAGACTGAAGCGTCCAAG	qPCR of endogenous *A. thaliana UBC21* house-keeping gene

Table S1. List and function of primers used in this study.

Dataset S1 (separate file). MurA and EPSPS amino acid sequences used in phylogenetic analysis

Dataset S2 (separate file). Trimmed multiple sequence alignment used to generate Maximum Likelihood phylogenetic tree

SI References

1. Guindon, S. *et al.* New Algorithms and Methods to Estimate Maximum-Likelihood Phylogenies: Assessing the Performance of PhyML 3.0. *Syst Biol* **59**, 307–321 (2010).
2. Soltis, D. E. *et al.* Angiosperm phylogeny: 17 genes, 640 taxa. *Am J Bot* **98**, 704–730 (2011).
3. Hu, H., Sun, P., Yang, Y., Ma, J. & Liu, J. Genome-scale angiosperm phylogenies based on nuclear, plastome, and mitochondrial datasets. *J Integr Plant Biol* **65**, 1479–1489 (2023).
4. López-Martínez, A. M. *et al.* Integrating Fossil Flowers into the Angiosperm Phylogeny Using Molecular and Morphological Evidence. *Syst Biol* **72**, 837–855 (2023).
5. Janssens, S. B. *et al.* A large-scale species level dated angiosperm phylogeny for evolutionary and ecological analyses. *Biodivers Data J* **8**, e39677 (2020).
6. Zeng, L. *et al.* Resolution of deep angiosperm phylogeny using conserved nuclear genes and estimates of early divergence times. *Nat Commun* **5**, 4956 (2014).
7. Group, T. A. P. *et al.* An update of the Angiosperm Phylogeny Group classification for the orders and families of flowering plants: APG IV. *Botanical Journal of the Linnean Society* **181**, 1–20 (2016).
8. Xu, X. *et al.* Genome sequence and analysis of the tuber crop potato. *Nature* **475**, 189–195 (2011).
9. Brockmöller, T. *et al.* Nicotiana attenuata Data Hub (NaDH): An integrative platform for exploring genomic, transcriptomic and metabolomic data in wild tobacco. *BMC Genomics* **18**, 1–11 (2017).
